# The relationship between language features and PTSD symptoms: a systematic review and meta-analysis

**DOI:** 10.3389/fpsyt.2025.1476978

**Published:** 2025-03-31

**Authors:** Zhenyuan Yu, Zixin Gu, Yonghong Shen, Jingbo Lu

**Affiliations:** Yueyang Hospital of Integrated Traditional Chinese and Western Medicine, Shanghai University of Traditional Chinese Medicine, Shanghai, China

**Keywords:** language features, post-traumatic stress disorder, meta-analysis, trauma, rapid screening

## Abstract

**Objective:**

The aim of this study is to investigate the relationship between language features and symptoms of Post-Traumatic Stress Disorder (PTSD) to determine if language features can serve as a reliable index for rapid screening and assessing PTSD.

**Methods:**

A comprehensive literature search was performed using Pubmed, Embase, Cochrane Central Register of Controlled Trials, Web of Science, and Ovid databases, augmented by backward reference tracking, to gather pertinent literature concerning language features and traumatic stress disorders published until August 2024.

**Results:**

Twelve observational studies were included, comprising a cumulative sample size of 5,706 cases. Various language analysis tools, such as Linguistic Inquiry and Word Count (LIWC), manual coding, and machine learning techniques, were employed in the studies. Meta-analysis findings revealed a positive correlation between death-related words and PTSD symptoms (OR 1.32, 95%CI 1.10 to 1.59, I² 79.4%, *p = 0.004*), as well as significant positive correlations between negative emotion words and PTSD symptoms (OR 1.21, 95%CI 1.11 to 1.32, I² 30.5%, *p < 0.001*), anger-related words and PTSD symptoms (OR 1.14, 95%CI 1.11 to 1.17, I² 0.0%, *p < 0.001*), word count and PTSD symptoms (OR 1.20, 95%CI 1.09 to 1.31, I² 11.2%, *p < 0.001*). Additionally, a positive correlation was observed between body-related words and hyperarousal symptoms of PTSD (OR 1.26, 95%CI 1.15 to 1.37, I² 0.0%, *p < 0.001*), intrusive symptoms (OR 1.40, 95%CI 1.16 to 1.68, I² 0.0%, *p < 0.001*), and avoidance symptoms (OR1.29, 95%CI 1.21 to 1.37, I² 0.0%, *p < 0.001*). Death-related words (OR 1.16, 95% CI 1.08 to 1.25, I² 0.0%, *p < 0.001*) and word count (OR 1.18, 95% CI 1.10 to 1.27, I² 0.0%, *p < 0.001*) were observed positive correlations between intrusive symptoms of PTSD. Conversely, no correlation was found between the use of words related to sadness, anxiety, positive emotions, first-person pronouns, sensory, cognitive-related words and PTSD symptoms.

**Conclusion:**

Death-related words, anger-related words, negative emotion words, body-related words and word count in Language features hold promise as a reliable indicator for rapid screening and assessing PTSD; however, further research is warranted to investigate their relationship with PTSD symptoms across various cultural contexts, genders, and types of trauma.

**Systematic Review Registration:**

https://www.crd.york.ac.uk/PROSPERO, identifier CRD42024528621.

## 1 Introduction

Post-Traumatic Stress Disorder (PTSD) is a severe, chronic, and potentially disabling condition that arises following exposure to or witnessing of traumatic events threatening an individual’s or others’ lives (1). Those who develop PTSD frequently exhibit symptoms such as intrusion, avoidance, and hyperarousal (2). Statistics indicate that approximately 70.4% of individuals experience significant trauma exposure during their lifetime, with an average of 2 types of trauma and 4.6 traumatic exposures (3). However, only a minority develop PTSD, with a prevalence of approximately 14% in the general population, 24% among minors (4), and 10-30% in combat veterans (5). In recent years, the impact of COVID-19 has exacerbated the already challenging working conditions for healthcare workers, characterized by heavy shift work, sleep deprivation, substantial responsibility for critically ill and severely traumatized patients, and continuous exposure to patient deaths and suffering. Consequently, there has been a notable increase from 10.73% to 20.84% in the incidence of PTSD among healthcare workers (6). However, among individuals who have witnessed traumatic events, only 5% receive diagnoses of trauma-related disorders in electronic health records (7), and the time from trauma to diagnosis can exceed 586 days (8). Therefore, rapidly and effectively screening individuals who may suffer from trauma-related conditions is crucial for providing timely treatment and preventing other adverse outcomes (9, 10).

Traditional assessment and diagnosis of PTSD typically employ two main methods: interviews and scales (11). Semi-structured diagnostic interviews, such as the Clinician-Administered PTSD Scale for DSM-5 (CAPS-5), are regarded as some of the most effective and comprehensive tools for diagnosing PTSD (12). These interviews can thoroughly capture the severity and functional impairment of PTSD (13). However, their complexity and time-consuming nature limit their widespread application (12, 14). For patients requiring multiple assessments, prolonged interviews can increase the burden and potentially lead to resistance, which may compromise the accuracy and consistency of the evaluation (11). Additionally, because this method relies on clinical interviews (15), it remains challenging for individuals who are busy, such as healthcare workers, or those who tend to deny or suppress emotional distress. Self-report scales, such as the Posttraumatic Stress Disorder Checklist for DSM-5 (PCL-5), although convenient and not requiring professional guidance, are easily influenced by subjective factors, potentially leading to inaccurate assessment results (16), for example, individuals’ emotional state, cultural background, and social expectations may cause deviations in the severity of their reported symptoms (13). Moreover, the fear of social stigmatization often prevents these individuals from seeking psychological support, causing them to conceal their true emotions and potentially worsening their condition (17). Other scales, such as the Impact of Event Scale-Revised (IES-R), can be used to assess PTSD symptoms but lack comprehensive coverage, rendering them unsuitable as diagnostic tools (18, 19).

One promising approach to improving the screening process for trauma-related disorders, as indicated by previous research, is the use of quantitative analysis based on language features (20). Language features exhibited by individuals are believed to provide key insights into their emotional, physical, and mental states (21). Numerous studies have investigated language styles and analyzed specific language features or vocabulary usage (22–24). These features may include the proportion of words that naturally refer to specific topics in the data, as well as features reflecting emotional, social, and cognitive processes obtained using existing dictionaries (25). In recent years, an increasing number of studies have explored the relationship between language features and PTSD. These studies have found that the use of negative emotion words, cognitive words, death-related words, anxiety-related words, and pronouns differ between PTSD patients and healthy individuals, with the correlations between these language features and PTSD symptoms being more specific than other mental health disorders (26–37). For example, the frequent occurrence of negative emotion words may reflect an individual’s persistent emotional distress (34), while the use of death-related words may indicate the intense memory and emotional response to trauma (32). Additionally, some studies have found a higher proportion of cognitive vocabulary in the language of PTSD patients, suggesting that they may experience a higher cognitive load or confusion when processing trauma-related memories (28, 29). Due to differences in analytical approaches, existing conclusions often lack consistency. For instance, the LIWC (Linguistic Inquiry and Word Count) tool systematically analyzes text using a fixed dictionary, enabling efficient assessment of emotional, cognitive, and social features (38). However, its reliance on predefined categories limits its ability to capture contextual nuances, such as sarcasm or metaphors (39, 40). In contrast, manual coding offers more flexibility, allowing researchers to interpret language features within specific contexts (41). Yet, it is more subjective, reliant on the coder’s judgment, and less efficient when applied to large datasets (41, 42). Additionally, due to differences in study subject selection, timing, scale usage, the existing conclusions lack consistency. For instance, regarding word count, some studies have found that participants who used more words in trauma narratives reported fewer PTSD symptoms (32, 43), while others have found no association between these two variables (27, 28). Similarly, while some studies have observed that PTSD patients use more death-related words (32, 34), another study found that PTSD patients used fewer death-related words (31).

The analysis of language features not only helps characterize the symptoms of PTSD but can also aids in early detection and screening. In particular, with the continuous advancement of artificial intelligence (AI) and machine learning technologies, AI-based language analysis methods can efficiently identify language features and reduce self-reporting biases (37). By analyzing large amounts of language data, these technologies can automatically extract language patterns related to emotions, cognition, and social factors from texts, helping researchers and clinicians more accurately identify early signs of PTSD symptoms (44). However, the drawbacks of these technological methods include their potential failure to fully consider context and non-explicit emotions and the need for high-quality annotated data to ensure the accuracy of the models (45). In comparison, traditional interview methods, although they can provide detailed and in-depth analyses of language behaviors (46), capturing subtle emotional and cognitive differences, are often limited by the subjective judgment of the analyst and require significant time and effort (47, 48).

Existing research has adopted various methods to focus on different aspects of language and PTSD. We hypothesize that death-related words, negative emotion words, and body-related words in language features are closely associated with PTSD and reflect different PTSD symptom profiles. Therefore, this meta-analysis aims to include all relevant studies, cover different dimensions of language features, and clarify whether language features can serve as reliable indicators for the rapid screening of post-traumatic stress disorder and improving the screening process for trauma-related disorders.

## 2 Materials and methods

### 2.1 Search strategy

The researchers in this study systematically searched five electronic databases (PubMed, Embase, Cochrane Central Register of Controlled Trials, Web of Science, and Ovid) from their inception to August 2024. The search strategy was structured according to the PECOS framework: (P) Population—individuals who have undergone a traumatic event and are diagnosed with PTSD; (E) Exposure—kinds of traumatic events; (C) Comparator—individuals devoid of PTSD symptoms; (O) Outcomes—the association between language features and PTSD symptoms; (S) Study type—observational studies. The detailed search strategy is presented in **Table 1** (PubMed is provided as an exemplar; similar search terms were adapted for other databases). The review was registered in PROSPERO under the code CRD42024528621.

**Table 1:** Search strategy on PubMed.

#1	Stress Disorders, Post-Traumatic[MeSH Terms]
#2	((((((((((((((((((((((Stress Disorders, Post-Traumatic) OR (Post-Traumatic Stress Disorder)) OR (Stress Disorder, Post-Traumatic)) OR (Neuroses, Post-Traumatic)) OR (Neuroses, Post Traumatic)) OR (Post-Traumatic Neuroses)) OR (PTSD)) OR (Neuroses, Posttraumatic)) OR (Posttraumatic Neuroses)) OR (Post-Traumatic Stress Disorders)) OR (Post Traumatic Stress Disorders)) OR (Posttraumatic Stress Disorders)) OR (Posttraumatic Stress Disorder)) OR (Stress Disorder, Posttraumatic)) OR (Stress Disorders, Posttraumatic)) OR (Post Traumatic Stress Disorder)) OR (Stress Disorder, Post Traumatic)) OR (Delayed Onset Post-Traumatic Stress Disorder)) OR (Delayed Onset Post Traumatic Stress Disorder)) OR (Chronic Post-Traumatic Stress Disorder)) OR (Chronic Post Traumatic Stress Disorder)) OR (Acute Post-Traumatic Stress Disorder)) OR (Acute Post Traumatic Stress Disorder)
#3	(#1) OR (#2)
#4	((((((((Language features) OR (Language feature)) OR (Linguistic features)) OR (linguistic feature)) OR (linguistic characteristics)) OR (linguistic characteristics)) OR (Speech analysis)) OR (Voice analysis)) OR (speech-based assessment)
#5	(#3) AND (#4)

### 2.2 Inclusion criteria

(1) The study included individuals who have experienced traumatic events and whose symptoms meet the diagnostic criteria for PTSD. (2) Language analysis was performed on verbal or written language materials, using methods such as sentiment analysis, lexical analysis, grammar analysis, or automated tools like Natural Language Processing. (3) The study provided quantitative data on the relationship between language features and the severity or frequency of PTSD symptoms.

### 2.3 Exclusion criteria

(1) Absence of correlational data, (2) Participants lacking PTSD symptoms or presenting other mental disorders, (3) Language analysis employed exclusively for intervention purposes without exploring its association with PTSD symptoms, (4) Case studies, reviews, or theoretical papers devoid of primary data.

### 2.4 Study selection

Literature screening and exclusion were performed using the literature management software EndNote. Following the removal of duplicates, two researchers screened the titles of the literature for case studies, review papers, conference papers, protocols, and communications. Subsequently, they reviewed the abstracts to reaffirm the inclusion and exclusion criteria of the literature. Ultimately, the full texts of the remaining literature were examined to make final inclusion decisions. Throughout this process, two researchers independently screened the literature, and the consistency of their selections was evaluated using the Kappa coefficient. In cases of disagreement, a third researcher was involved in the decision-making process to ensure the accuracy of the literature selection.

### 2.5 Data extraction

Data from the included studies were documented in a ten-item data extraction table with the following categories: (1) Author, (2) Publication Year, (3) Country, (4) Sample Size, (5) Participants, (6) Language Analysis Techniques, (7) Text Source, (8) Language Features, (9) PTSD Diagnosis Method, and (10) PTSD Symptoms.

### 2.6 Risk of bias of individual studies

The quality of the included studies was evaluated by two independent researchers. To ensure consistency and reliability, cross-validation was used, and any disputes were resolved with the involvement of a third researcher. All assessors had backgrounds in epidemiology and statistics, ensuring the effectiveness of the evaluation process. All studies were evaluated using the Newcastle-Ottawa Scale (NOS) (49). The NOS evaluates the quality of non-randomized studies based on three broad perspectives: the selection of the study groups (0-4 points), the comparability of the groups (0-2 points), and the ascertainment of either the exposure or outcome of interest (0-3 points). A study can be awarded a maximum of one star for each numbered item within the Selection and Exposure categories, and a maximum of two stars can be given for Comparability. The total NOS score ranges from 0 to 9 points, with scores of ≤ 4 indicating low-quality studies, 5-6 indicating medium-quality studies, and ≥ 7 indicating high-quality studies.

### 2.7 Data analysis

In this study, focusing on the correlation between language characteristics and PTSD symptoms, we conducted a meta-analysis on studies that included two or more research articles, as referenced in the literature (50, 51). The meta-analysis was planned to assess the following seven aspects:1) The relationship between cognitive words and PTSD symptoms; 2) The relationship between death-related words and PTSD symptoms; 3) The relationship between emotion-related words and PTSD symptoms; 4) The relationship between first-person pronouns and PTSD symptoms; 5) The relationship between word count and PTSD symptoms; 6) The relationship between body-related words and PTSD symptoms; and 7) The relationship between sensory-related words and PTSD symptoms. The research team extracted pertinent data from the selected literature and utilized the metafor package in R language to compute the effect size (TE) and its standard error (SE). Subsequently, the meta-analysis was performed using Stata software (version 15.1). A forest plot was generated using Stata’s metan to visually illustrate the effect sizes and their 95% confidence intervals for the correlation coefficients between language characteristics and PTSD symptoms across various studies. Additionally, the heterogeneity among the included studies was comprehensively evaluated using the I² statistic and Q test for quantitative analysis. A random-effects model was employed for the meta-analysis to account for the heterogeneity among the included studies. This model assumes that the true effect sizes vary across studies and provides a more conservative estimate of the overall effect size compared to a fixed-effects model. The choice of the random-effects model was based on the expectation of clinical and methodological diversity among the studies, which is common in meta-analyses of observational studies (52). To investigate potential publication bias, Begg’s test and Egger’s test for metabias were performed.

## 3 Results

### 3.1 Study and identification and selection

**Figure 1:**
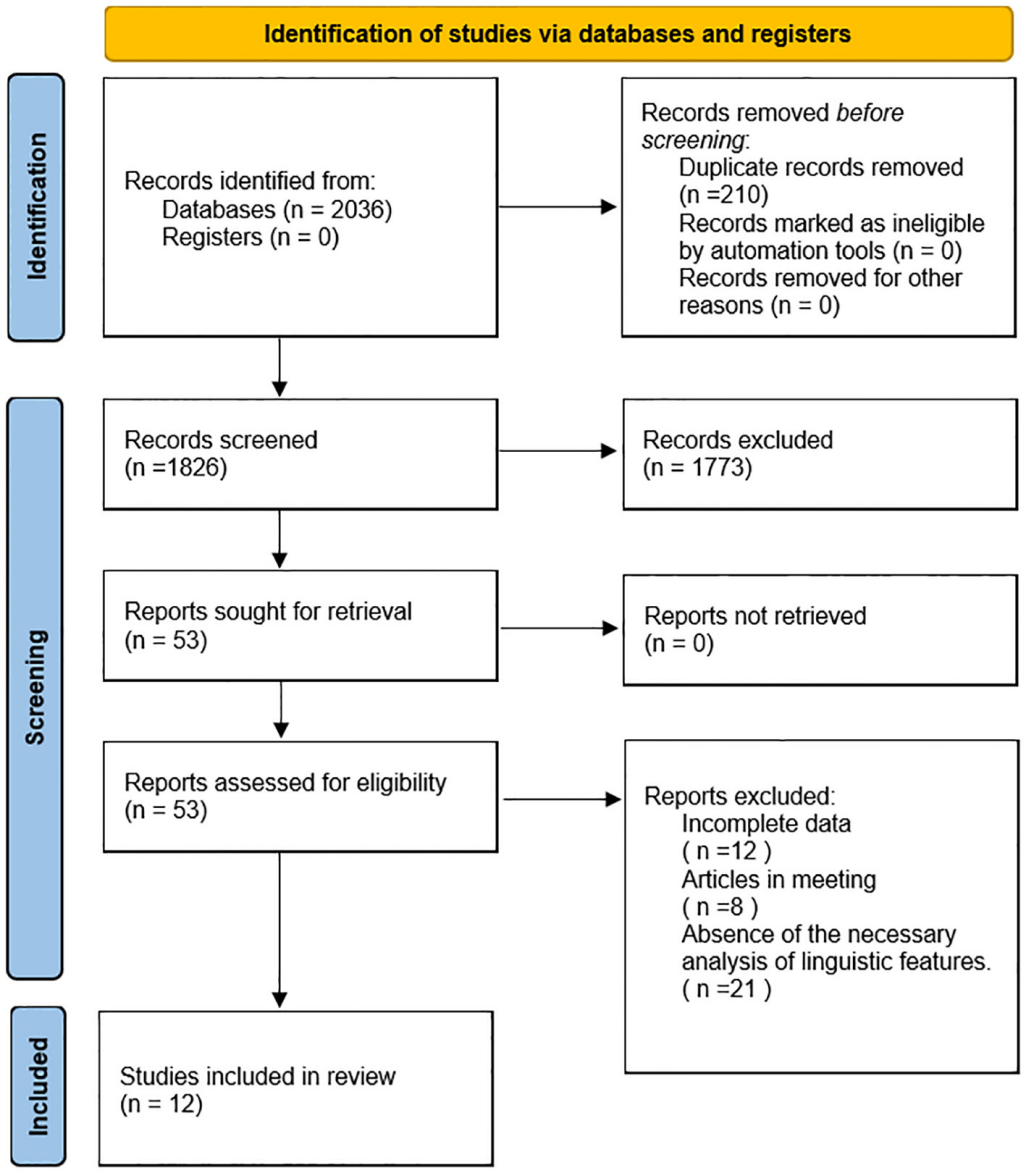
Process for the selection.

During the database retrieval process, a total of 2,036 documents were initially retrieved. After eliminating redundant documents, a total of 1,826 documents were included in the initial screening based on titles and abstracts, out of which 1,773documents were excluded for not meeting the inclusion criteria. Subsequently, a thorough review of the full-text was conducted on the remaining 53 documents after screening, leading to the exclusion of 41 documents due to issues such as data inaccessibility, the articles being conference reports, or the absence of the necessary analysis of linguistic features. Consequently, 12 documents met the inclusion criteria and were selected for the final synthesis. The consistency of the screening process was assessed using the Kappa coefficient, which yielded a value of 0.85, indicating high consistency. (Refer to **Figure 1** for the selection process.).

### 3.2 Quality assessment of the included studies

The Newcastle-Ottawa Scale (NOS) was employed to assess the quality of the 12 included studies, with scores ranging from 5 to 9. A majority of the studies achieved scores above 7, indicating predominantly high quality among the selected research, thereby promising a level of reliability in the results synthesized for this meta-analysis. Ultimately, based on the NOS criteria, eight studies were categorized as high-quality, while the remaining four were deemed of moderate quality. The distribution of these scores is detailed in **Supplementary Table 1**.

### 3.3 Characteristics of the included studies

The meta-analysis included 12 observational studies, involving a cohort of 5,706 patients diagnosed with PTSD following exposure to traumatic events. The research data were sourced from transcribed interview texts (n=7) and written texts (n=5). All 10 studies used LIWC for natural language processing, with two studies also employing machine learning. Only two studies used manual coding, one of which was combined with machine learning. These studies analyzed an array of language features within patient narratives, including the usage of cognitive words (7 studies), negative emotion words (4 studies), positive emotion words (3 studies), death-related words (7 studies), word count (6 studies), anxiety-related words (4 studies), first-person singular pronouns (4 studies), first-person plural pronouns (2 studies), anger-related words (3 studies), sadness-related words (2 studies), hearing-related words (2 studies), touching-related words (2 studies), seeing-related words (2 studies), and body-related words (3 studies). Concerning the assessment of PTSD symptoms, 7 studies designated them as the primary outcome measure, 4 studies examined PTSD symptoms alongside specific symptoms of intrusion, avoidance, and hyperarousal as outcome measures, and 1 study focused on intrusion, avoidance, and hyperarousal symptoms as outcome measures. The populations under study were diverse, including survivors of sexual assault (2 studies), domestic violence (1 study), the 9/11 attacks (1 study), genocide (1 study), natural disasters (1 study), COVID-19 (2 studies), the Paris terrorist attacks (1 study), and patients formally diagnosed with PTSD (3 studies). The characteristics details of these studies are cataloged in **Table 2**.

**Table 2:** Characteristics of the studies included in the meta-analysis.

Author	Country	Year	Sample size	Participants	Language Analysis Techniques	Text Source	Language features	PTSD measure	PTSD symptoms
Alvarez-Conrad	USA	2001	22	Female assault victims	LIWC	Interview texts	Cognitive words Negative emotion words Positive emotion words Death related Word count	PSS-I	PTSD symptoms
D’Andrea	USA	2012	28	College students who had relative to 9/11	LIWC	Trauma writing	Death related Word count Anxiety related First-person singular Anger related First-person plural	IES-R	PTSD symptoms Intrusion symptoms Avoidance symptoms Hyperarousal symptoms
Miragoli	Italy	2014	58	Victims of sexual abuse	LIWC	Interview texts	Cognitive words(Causal words) Negative emotion words Death related	DSM IV	PTSD symptoms
Ng	USA	2015	61	survivors of the 1994 Rwandan Genocide against the Tutsi	LIWC	Interview texts	Word count Anxiety related Anger related Sadness related Hearing related Touching related Seeing related Body related	IES-R	Intrusion symptoms Avoidance symptoms Hyperarousal symptoms
Papini	USA	2015	23	People who had at least moderately severe PTSD symptoms	LIWC	Interview texts	Cognitive words Death related Anxiety related Sadness related	DSM IV Clinician-Administered PTSD Scale	PTSD symptoms Intrusion symptoms Avoidance symptoms Hyperarousal symptoms
Marshall	USA	2022	55	Survivors of Hurricane Harvey	LIWC	Trauma writing	Cognitive words Death related Word count Hearing related Touching related Seeing related Body related	IES-R	PTSD symptoms Intrusion symptoms Avoidance symptoms Hyperarousal symptoms
Marengo	Netherlands	2022	5048	People who had experienced stressful events of COVID-19	LIWC and machine learning	Trauma writing	Negative emotion words Anger related	Global Psychotrauma Screen (GPS)	PTSD symptoms
Castiglioni	Itlay	2023	135	Healthcare workers during COVID-19	LIWC	Trauma writing	Cognitive words Negative emotion words Positive emotion words Death related First-person singular	Los Angeles Symptom Checklist (LASC)	PTSD symptoms
Ellis	USA	2023	33	People who had PTSD symptoms	LIWC	Trauma writing	Cognitive words(Causal words) Death related	DSM IV	PTSD symptoms
Frabetti	France	2023	19	Female victims of domestic violence	Manual coding	Interview texts	First-person singular	PCL-5	PTSD symptoms
Son	USA	2023	75	People who had PTSD symptoms	LIWC and machine learning	Interview texts	Word count Anxiety related First-person singular First-person plural	PCL-5	PTSD symptoms
Quillivic	France	2024	149	Survivors of terrorist attacks in Paris	Manual coding and machine learning	Interview texts	Death related Word count Body related Positive emotion words	DSM-5	PTSD symptoms Intrusion symptoms Avoidance symptoms Hyperarousal symptoms

### 3.4 Meta-analysis results between language features and PTSD symptoms

**Figure 2** shows all the meta-analysis results and specific details of meta-analysis results will be presented in **Supplementary Figures 1**-**39**. Each figure is labeled with the specific language feature and PTSD symptom being analyzed, and all figures are referenced in the text to facilitate understanding.

**Figure 2:**
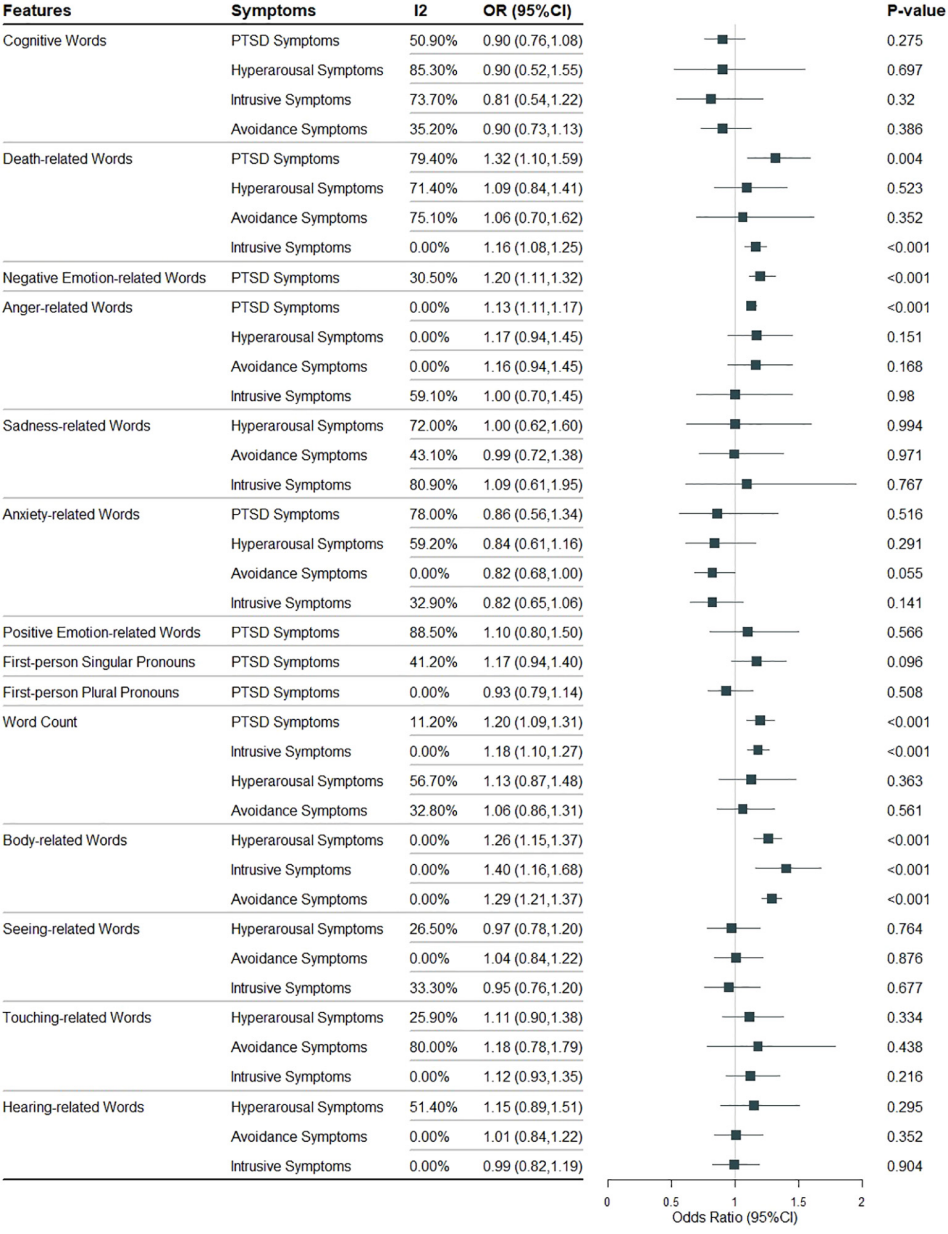
All the meta-analysis results of the relationship between different language features and PTSD symptoms.

#### 3.4.1 Relationship between cognitive words and PTSD symptoms

The use of cognitive words (6 studies, 321 cases) was not associated with PTSD symptoms (OR 0.90, 95% CI 0.76 to 1.08, I² 50.9%, *p = 0.275*) (**Supplementary Figure S1**). Similarly, no association was found with hyperarousal symptoms (OR 0.90, 95% CI 0.52 to 1.55, I² 85.3%, *p = 0.697*) or intrusive symptoms (OR 0.81, 95% CI 0.54 to 1.22, I² 73.7%, *p = 0.320*) across 3 studies with 106 cases (**Supplementary Figures S2, 3**). Additionally, no association was found with avoidance symptoms (OR 0.90, 95% CI 0.73 to 1.138, I² 35.2%, *p = 0.386*) in 4 studies with 139 cases (**Supplementary Figure S4**).

#### 3.4.2 Relationship between death-related words and PTSD symptoms

The use of death-related words (7 studies, 470 cases) was significantly associated PTSD symptoms (OR 1.32, 95% CI 1.10 to 1.59, I² 79.4%, *p = 0.004*) (**Supplementary Figure S5**). The use of death-related words (4 studies, 255 cases) was not significantly associated with hyperarousal symptoms (OR 1.09, 95% CI 0.84 to 1.41, I² 71.4%, *p = 0.523*) (**Supplementary Figure S6**), but a significant association was found with intrusive symptoms (OR 1.16, 95% CI 1.08 to 1.25, I² 0.0%, *p < 0.001*) (**Supplementary Figure S7**). The use of death-related words (3 studies, 106 cases) was not significantly associated with avoidance symptoms (OR 1.06, 95% CI 0.70 to 1.62, I² 75.1%, *p = 0.352*) (**Supplementary Figure S8**).

#### 3.4.3 Relationship between emotion-related words and PTSD symptoms

The use of negative emotion-related words (4 studies, 5236 cases) was significantly associated with PTSD symptoms (OR 1.21, 95% CI 1.11 to 1.32, I² 30.5%, *p < 0.001*) (**Supplementary Figure S9**). The use of anger-related words (2 studies, 5076 cases) was significantly associated with PTSD symptoms (OR 1.14, 95% CI 1.11 to 1.17, I² 0.0%, *p < 0.001*) (**Supplementary Figure S10**), but no association was found with hyperarousal symptoms (OR 1.17, 95% CI 0.94 to 1.45, I² 0.0%, *p = 0.151*) (**Supplementary Figure S11**), avoidance symptoms (OR 1.16, 95% CI 0.94 to 1.45, I² 0.0%, *p = 0.168*) (**Supplementary Figure S12**), or intrusive symptoms (OR 1.00, 95% CI 0.70 to 1.45, I² 59.1%, *p = 0.980*) across 2 studies with 89 cases (**Supplementary Figure S13**).

The use of sadness-related words (2 studies, 84 cases) was not associated with hyperarousal symptoms (OR 1.00, 95% CI 0.62 to 1.60, I² 72.0%, *p = 0.994*) (**Supplementary Figure S14**), avoidance symptoms (OR 0.99, 95% CI 0.72 to 1.38, I² 43.1%, *p = 0.971*) (**Supplementary Figure S15**), or intrusive symptoms (OR 1.09, 95% CI 0.61 to 1.95, I² 80.9%, *p = 0.767*) (**Supplementary Figure S16**).

The use of anxiety-related words (3 studies, 123 cases) was not associated with PTSD symptoms (OR 0.86, 95% CI 0.56 to 1.34, I² 79%, *p = 0.516*) (**Supplementary Figure S17**). Furthermore, no association was found with hyperarousal symptoms (OR 0.84, 95% CI 0.61 to 1.16, I² 59.2%, *p = 0.291*) (**Supplementary Figure S18**), avoidance symptoms (OR 0.83, 95% CI 0.68 to 1.00, I² 0.0%, *p = 0.055*) (**Supplementary Figure S19**), or intrusive symptoms (OR 0.83, 95% CI 0.65 to 1.06, I² 32.9%, *p = 0.141*) across 3 studies with 112 cases (**Supplementary Figure S20**).

The use of positive emotion-related words (3 studies, 306 cases) was not associated with PTSD symptoms (OR 1.10, 95% CI 0.80 to 1.50, I² 88.5%, *p = 0.566*) (**Supplementary Figure S21**).

#### 3.4.4 Relationship between first-person pronouns and PTSD symptoms

The use of first-person singular pronouns (4 studies, 257 cases) was not associated with PTSD symptoms (OR 1.17, 95% CI 0.97 to 1.40, I² 41.2%, *p = 0.096*) (**Supplementary Figure S22**). Similarly, the use of first-person plural pronouns (2 studies, 103 cases) was not associated with PTSD symptoms (OR 0.93, 95% CI 0.79 to 1.14, I² 0.0%, *p = 0.508*) (**Supplementary Figure S23**).

#### 3.4.5 Relationship between word count and PTSD symptoms

Word count (5 studies, 329 cases) was significantly associated with PTSD symptoms (OR 1.20, 95% CI 1.09 to 1.31, I² 11.2%, *p < 0.001*) (**Supplementary Figure S24**). Word count (4 studies, 293 cases) was also significantly associated with intrusive symptoms of PTSD (OR 1.18, 95% CI 1.10 to 1.27, I² 0.0%, *p < 0.001*) (**Supplementary Figure S25**). However, no significant associations were found between word count (3 studies, 144 cases) and hyperarousal symptoms (OR 1.13, 95% CI 0.87 to 1.48, I² 56.7%, *p = 0.363*) or avoidance symptoms (OR 1.06, 95% CI 0.86 to 1.31, I² 32.8%, *p = 0.561*) (**Supplementary Figures S26**, **27**).

#### 3.4.6 Relationship between body-related words and PTSD symptoms

The use of body-related words (3 studies, 265 cases) was significantly associated with hyperarousal symptoms (OR 1.26, 95% CI 1.51 to 1.37, I² 0.0%, *p < 0.001*) (**Supplementary Figure S28**), avoidance symptoms (OR 1.29, 95% CI 1.21 to 1.37, I² 0.0%, *p < 0.001*) (**Supplementary Figure S29**), and intrusive symptoms (OR 1.40, 95% CI 1.16 to 1.68, I² 0.0%, *p < 0.001*) across 2 studies with 116 cases (**Supplementary Figure S30**).

#### 3.4.7 Relationship between sensory words and PTSD symptoms

The use of seeing-related words (2 studies, 116 cases) was not associated with hyperarousal symptoms (OR 0.97, 95% CI 0.78 to 1.20, I² 26.5%, *p = 0.764*) (**Supplementary Figure S31**), avoidance symptoms (OR 1.01, 95% CI 0.84 to 1.22, I² 0.0%, *p = 0.876*) (**Supplementary Figure S32**), or intrusive symptoms (OR 0.95, 95% CI 0.76 to 1.20, I² 33.3%, *p = 0.677*) (**Supplementary Figure S33**).

The use of touching-related words (2 studies, 116 cases) showed no association with symptoms of hyperarousal (OR 1.12, 95% CI 0.90 to 1.38, I² 25.9%, *p = 0.334*) (**Supplementary Figure S34**), avoidance symptoms (OR 1.19, 95% CI 0.78 to 1.79, I² 80.0%, *p = 0.438*) (**Supplementary Figure S35**), or intrusive symptoms (OR 1.13, 95% CI 0.93 to 1.35, I² 0.0%, *p = 0.216*) (**Supplementary Figure S36**).

Similarly, the use of hearing-related words (2 studies, 116 cases) was not associated with symptoms of hyperarousal (OR 1.15, 95% CI 0.89 to 1.51, I² 51.4%, *p = 0.295*) (**Supplementary Figure S37**), avoidance symptoms (OR 1.01, 95% CI 0.84 to 1.22, I² 0.0%, *p = 0.352*) (**Supplementary Figure S38**), or intrusive symptoms (OR 0.99, 95% CI 0.82 to 1.19, I² 0.0%, *p = 0.904*) (**Supplementary Figure S39**).

### 3.5 Publication bias test

Begg’s test and Egger’s test were performed for all results to assess potential publication bias. Both tests indicated no significant evidence of publication bias (*p > 0.05*), suggesting that the literature included in this study was not substantially affected by publication bias.

## 4 Discussion

This systematic review and meta-analysis aimed to systematically compare the relationship between language features and PTSD symptoms to determine whether specific language features are associated with PTSD symptoms, and to evaluating the potential of language features as reliable markers for PTSD. Our findings indicate that the use of death-related words, negative emotion words, anger-related words, body-related words, and word count is significantly positively correlated with PTSD symptoms. Additionally, the use of death-related words and word count is also associated with intrusive PTSD symptoms. No significant associations were observed between the use of words related to sadness, anger, anxiety, positive emotions, first-person pronouns, sensory and cognitive-related words and PTSD symptoms.

Our meta-analysis of seven studies highlighted a positive correlation between the use of death-related words and PTSD symptoms, aligning with findings from Alvarez-Conrad, D’Andrea, Miragoli, Marshall, Castiglioni, and Ellis (27–29, 32, 34, 35). This correlation likely reflects the intrinsic link between PTSD and life-threatening experiences, with death-related language serving as a reflection of the trauma’s nature and severity. When describing trauma-related memories and feelings, individuals more frequently use death-related vocabulary, expressing intense concern and fear for their safety and questioning the nature of security (35, 53). This is also associated with intrusive symptoms. Contrary to Papini (31), who observed a negative correlation yet acknowledged death-related words as a fundamental expression of PTSD, our study’s findings suggest that for individuals on a path toward recovery, engaging with death-related vocabulary might be indicative of processing trauma rather than avoiding it. This interpretation underscores the complexity of trauma-related language patterns and their relationship with the PTSD symptomatology.

The analysis further supports that the use of emotion words, especially those expressing negative emotions and anger, is significant positively correlated with PTSD symptoms. This is consistent with D’Andrea, Miragoli, Ng, Castiglioni, and Ellis (28–30, 34, 35), reflecting the prevalent emotional regulation challenges in PTSD sufferers and their attempts to articulate traumatic experiences and emotional turmoil (54, 55). Anger, in particular, may directly relate to the regulation difficulties (56, 57), often manifesting in responses of hostility and anger to traumatic recollections, rather than denoting specific PTSD symptom dimensions (55). Interestingly, Alvarez-Conrad (27) identified a negative correlation between negative emotion word use and PTSD symptoms, possibly because the study sample included narratives from individuals undergoing narrative exposure therapy—a technique where confronting and processing avoided trauma memories and emotions reduces their negative impact (58–60). This suggests that individuals with chronic PTSD might be in the process of actively working through traumatic memories, rather than merely avoiding or suppressing negative emotions (61).

In comparison, other emotion words such as sadness, anxiety, and positive emotions did not demonstrate associations in this study. These findings are inconsistent with Alvarez‐Conrad, D’Andrea, Ng, Son (27, 28, 30, 31, 37), which may stem from anxiety and sadness being general emotional states that are not limited to PTSD patients (62–64), and some PTSD individuals may use positive emotion regulation strategies to cope with anxiety and sadness (65). Therefore, the use of sadness, anxiety, and positive emotion words alone might not comprehensively capture the actual PTSD symptomatology. At the same time, the associations between these emotional words and PTSD symptoms could be affected by various factors, such as sample characteristics, measurement tools, and individual differences, which could contribute to the lack of significant associations in our study.

In examining the relationship between word count and PTSD symptoms, we found a positive correlation, consistent with the findings of Alvarez‐Conrad, D’Andrea, Marshall, and Son (23, 24, 28, 33), suggesting that when describing traumatic experiences, individuals may use more words to express these intense and complex emotions (53).This level of detail may also be positively correlated with the severity of intrusive symptoms (66), which could explain the observed association between word count and both PTSD and intrusive symptoms. However, we did not observe an association between word count and PTSD symptoms of hyperarousal and avoidance. This result was not supported by Miragoli, Ng, and Marshall (29, 30, 32), possibly indicating that hyperarousal and avoidance symptoms may be less dependent on detailed narration of the traumatic event. For example, hyperarousal may be more reflected in the individual’s over-reaction to the current environment, and avoidance symptoms may be manifested in the intentional avoidance of trauma-related people, places, activities, or thoughts. The expression of these symptoms might not necessarily require the use of a large number of words due to individual differences (67).

We also found a positive correlation between the use of body-related words and the hyperarousal, intrusion, and avoidance symptoms of PTSD patients, as supported by the findings of Beaudreau, Ng, and Marshall (30, 32, 43), revealing the particular sensitivity of PTSD individuals to bodily sensations. This sensitivity may stem from the sustained physiological activation during a state of hyperarousal or serve as a psychological avoidance mechanism, prompting individuals to more frequently cite content related to body status in their language expression (68). Furthermore, research in the neurobiology of PTSD has identified associations between PTSD and abnormal activity in brain regions involved in processing bodily sensations and emotions, such as the amygdala, anterior cingulate cortex, and temporo-parietal junction (69–72). These neurobiological changes may result in significant differences in how patients process and express information related to body status, a phenomenon further reflected in their language use patterns, thereby providing additional support for our findings.

In our study, we did not find any associations with the use of first-person pronouns, whether singular or plural, contrary to the findings of D’Andrea, Castiglioni, Frabetti, and Son (28, 34, 36, 37). We posit that the use of first-person pronouns may not only reflect the narrator’s level of self-involvement but also be influenced by the context, purpose, and audience of the narration (73, 74). In traumatic narratives, even when expressing personal experiences and feelings, narrators may adjust their language style, including the use of first-person pronouns, based on the specific purpose of the narration (e.g., seeking support, empathy, or understanding), potentially influencing the detection of the relationship between the use of first-person pronouns and PTSD symptoms.

However, regarding the use of sensory-related words, although Ng and Marshall (30, 32) found different associations, our study did not reveal any associations between the use of visual, auditory, or tactile-related words and PTSD avoidance symptoms, intrusion symptoms, or hyperarousal symptoms. Nevertheless, this absence of associations does not diminish the importance of these sensory dimensions in PTSD. Conversely, this observation may suggest that the language expression of PTSD patients could be more complex and diverse across various sensory dimensions, or that the influence of these dimensions might be overshadowed by other more significant linguistic features. Moreover, these conclusions stem from data from only two studies, which might constrain statistical power and hinder the detection of significant associations.

Additionally, no associations were observed between cognitive words and PTSD symptoms, intrusive symptoms, avoidance symptoms, or hyperarousal symptoms. This contrasts with the findings of Alvarez‐Conrad, D’Andrea, Miragoli, Papini, Marshall, Castiglioni, and Ellis (27–29, 31, 32, 34, 35), and we posit that in PTSD patients, these cognitive processes may become exceedingly intricate, potentially leading to a disconnect between their cognitive processes and their language expression. For instance, individuals may experience profound feelings of self-blame or fear of the future, yet may find themselves unable or unwilling to articulate specific words that reflect these complex cognitive processes in their verbal expression (61). It has also been suggested that the predominant feature in the narrative is perceptual details rather than cognitive process words, which are more closely associated with the severity of trauma symptoms. This is because individuals may struggle to understand the traumatic event and therefore rely on sensory details rather than causal and insight words to describe it (75).

In summary, the use of death-related words, negative emotion words, anger-related words, and body-related words, as well as a higher word count, can serve as reliable indicators for the rapid screening and assessment of PTSD, aiding in the early clinical identification of high-risk individuals, especially in initial screenings or resource-limited settings. During treatment, monitoring changes in these language features may reflect treatment effectiveness. A decrease in the frequency of these words may reflect the patient’s adaptation to the traumatic experience and improvement in emotional state, with reductions in death-related words and word count specifically reflecting improvements in intrusive symptoms. This approach provides real-time feedback on treatment outcomes, reduces the burden and resistance associated with prolonged interviews, and enhances the accuracy and consistency of assessments, ultimately contributing to achieving optimal treatment results.

## 5 Strengths and limitations

This meta-analysis draws data from a variety of studies and provides a comprehensive examination of the relationship between language features and post-traumatic stress disorder (PTSD) symptoms. Our findings lay the groundwork for using language features as reliable markers for PTSD, thereby contributing to the early identification and treatment of the disorder.

Although a comprehensive search across major databases was conducted, some relevant studies may have been overlooked. The small number of studies included in this meta-analysis (only 12) may limit the generalizability of the results and weaken the conclusions. Furthermore, significant differences in participant characteristics (such as age, gender, cultural background, trauma types, and PTSD measurement tools) could introduce bias and limit the applicability of the findings to different populations. While no publication bias was detected, we acknowledge the possibility that it may exist, potentially leading to an overrepresentation of positive findings.

## 6 Conclusion

This systematic review and meta-analysis explored the relationship between language features and PTSD symptoms. The results indicate that death-related words, negative emotion words, and body-related words, and a higher word count are reliable indicators for the rapid screening and assessment of PTSD. Furthermore, reductions in death-related words and word count reflect improvements in intrusive symptoms. However, it is important to note that the current body of research is limited, with relatively small sample sizes and considerable variability in study designs. Future studies with larger, more diverse samples are needed to improve the generalizability of these findings. Exploring the relationship between language features and PTSD symptoms across cultures, genders, and trauma types is crucial for enhancing the cross-cultural applicability of the research. Longitudinal studies are also required to track changes in language over time, particularly during trauma recovery or symptom exacerbation. Real-time monitoring of language could improve the assessment of treatment outcomes, providing clinicians with valuable feedback for more precise diagnosis and intervention timing.

## Data Availability

The original contributions presented in the study are included in the article/**Supplementary Material**. Further inquiries can be directed to the corresponding author.
