# Supplementary material for: The relationship between language features and PTSD symptoms: a systematic review and meta-analysis
Source: Front Psychiatry. 2025 Mar 31;16:1476978. doi: 10.3389/fpsyt.2025.1476978 (PMC11994430; doi:10.3389/fpsyt.2025.1476978)
Supplement: Supplementary file 1 [file DataSheet1.pdf]

Supplementary Material

1 Supplementary Figures and Tables

1.1 Supplementary Figures

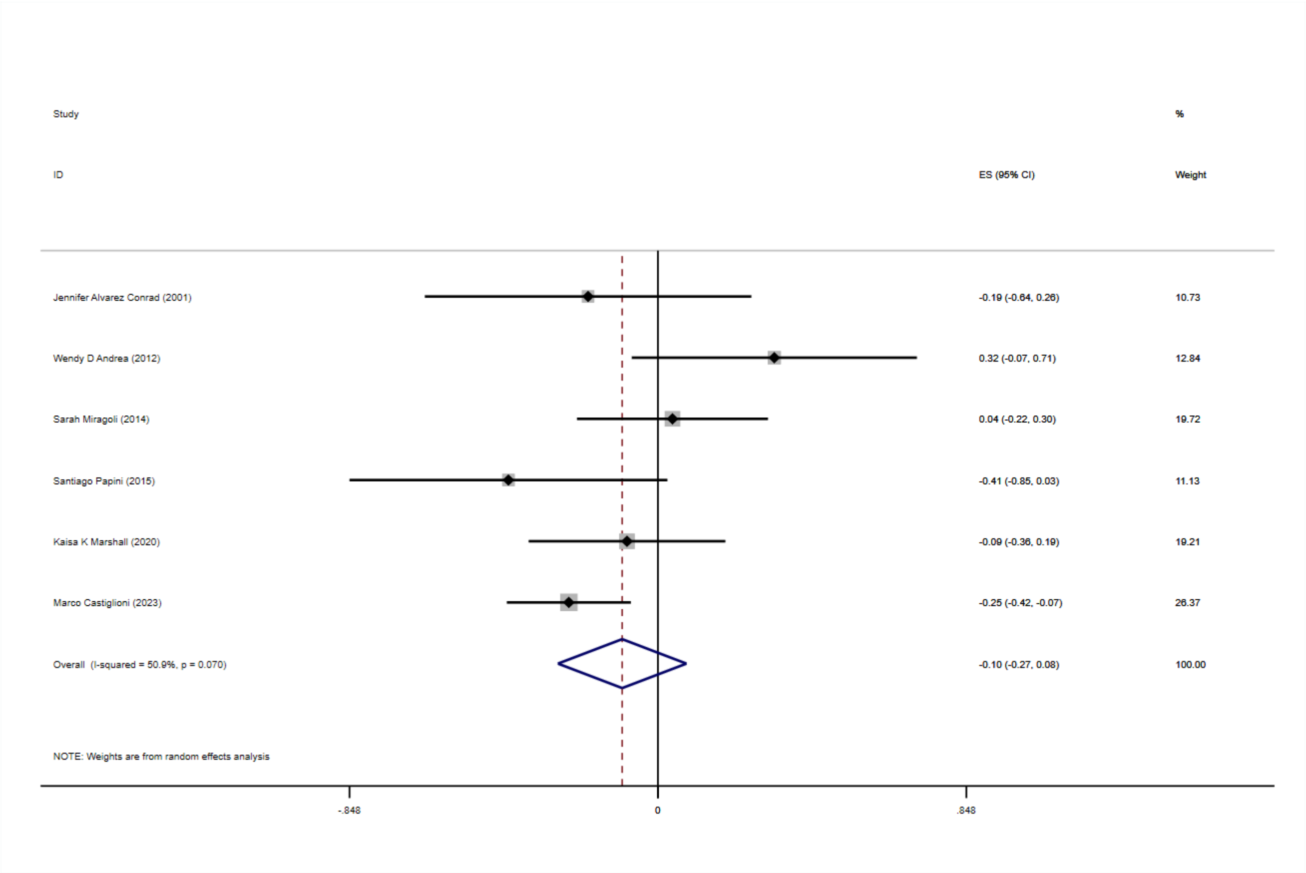

Supplementary Figure 1. cognitive words and PTSD symptoms

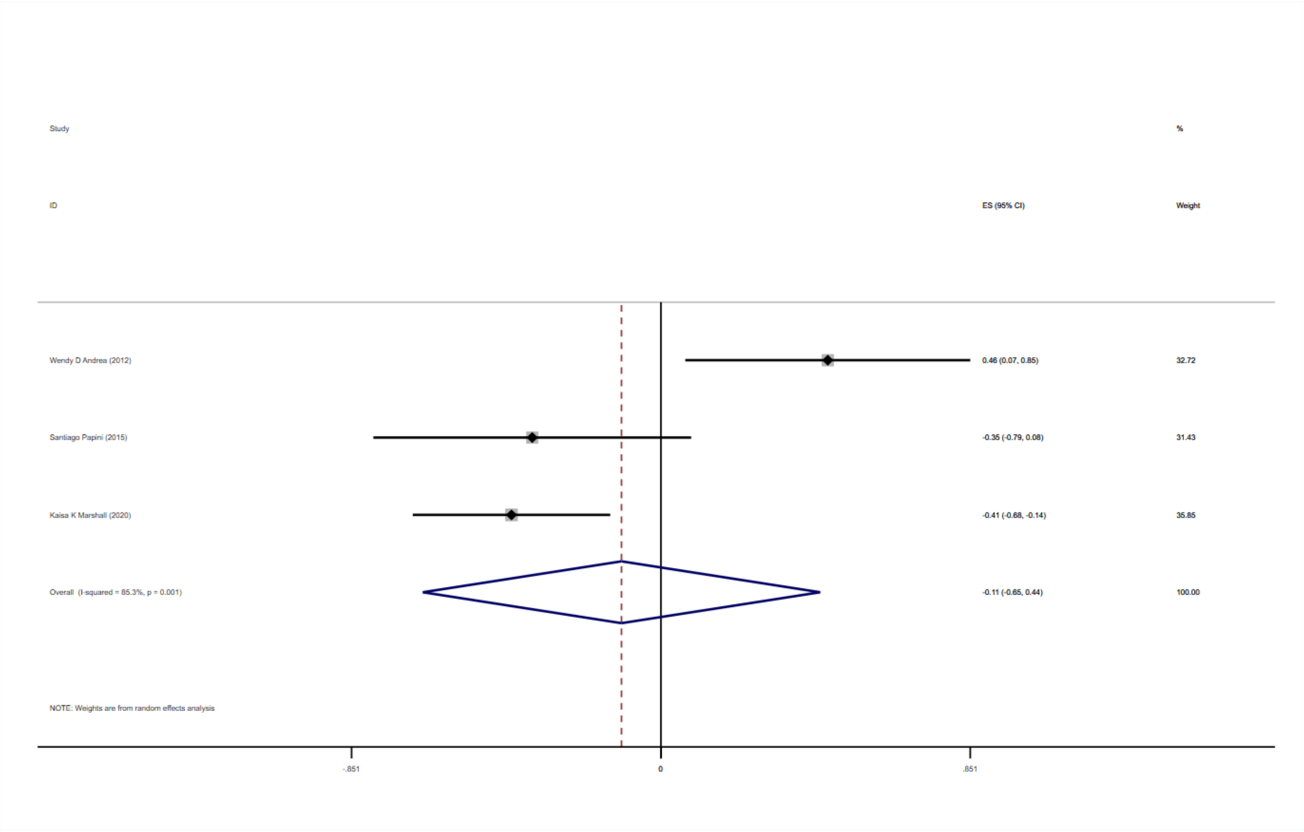

Supplementary Figure 2. cognitive words and hyperarousal symptoms

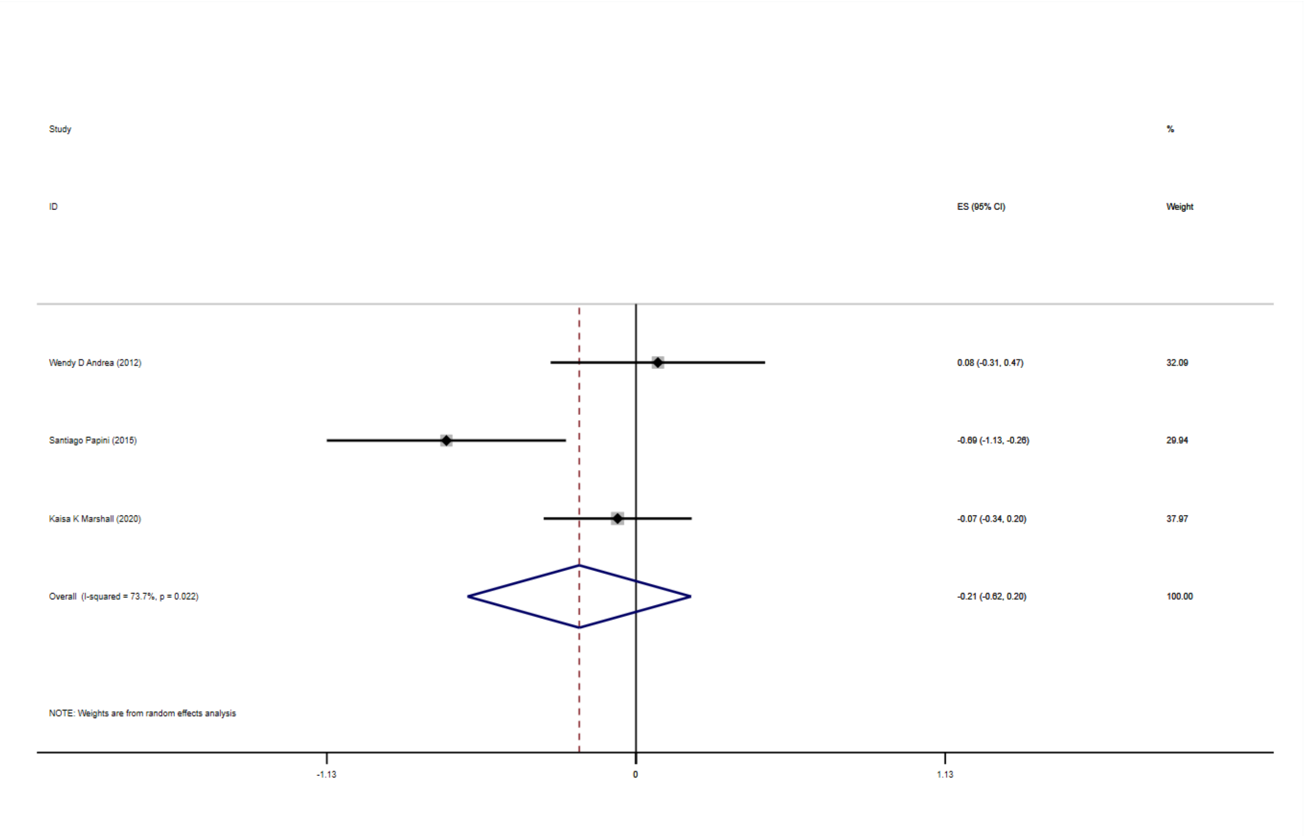

Supplementary Figure 3. cognitive words and intrusive symptoms

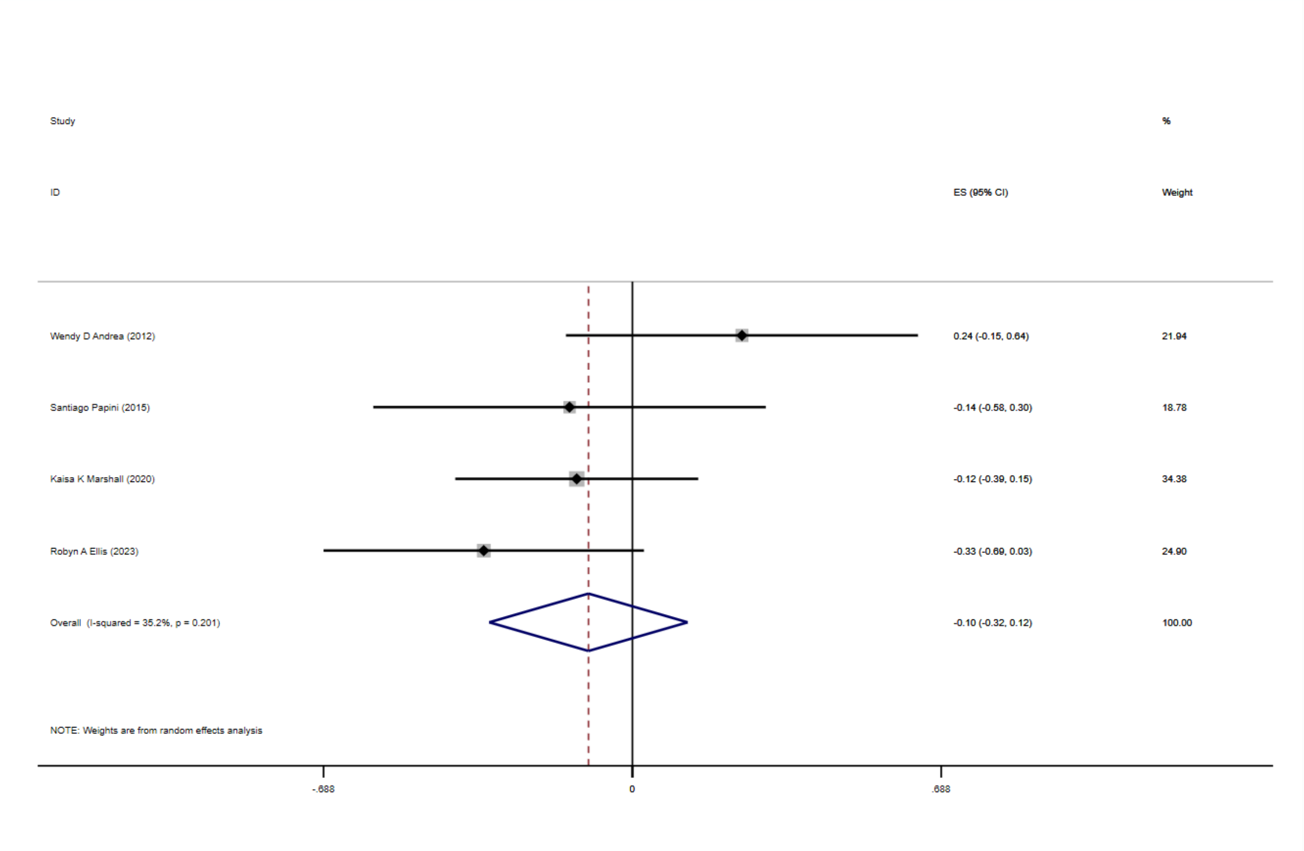

Supplementary Figure 4. cognitive words and avoidance symptoms

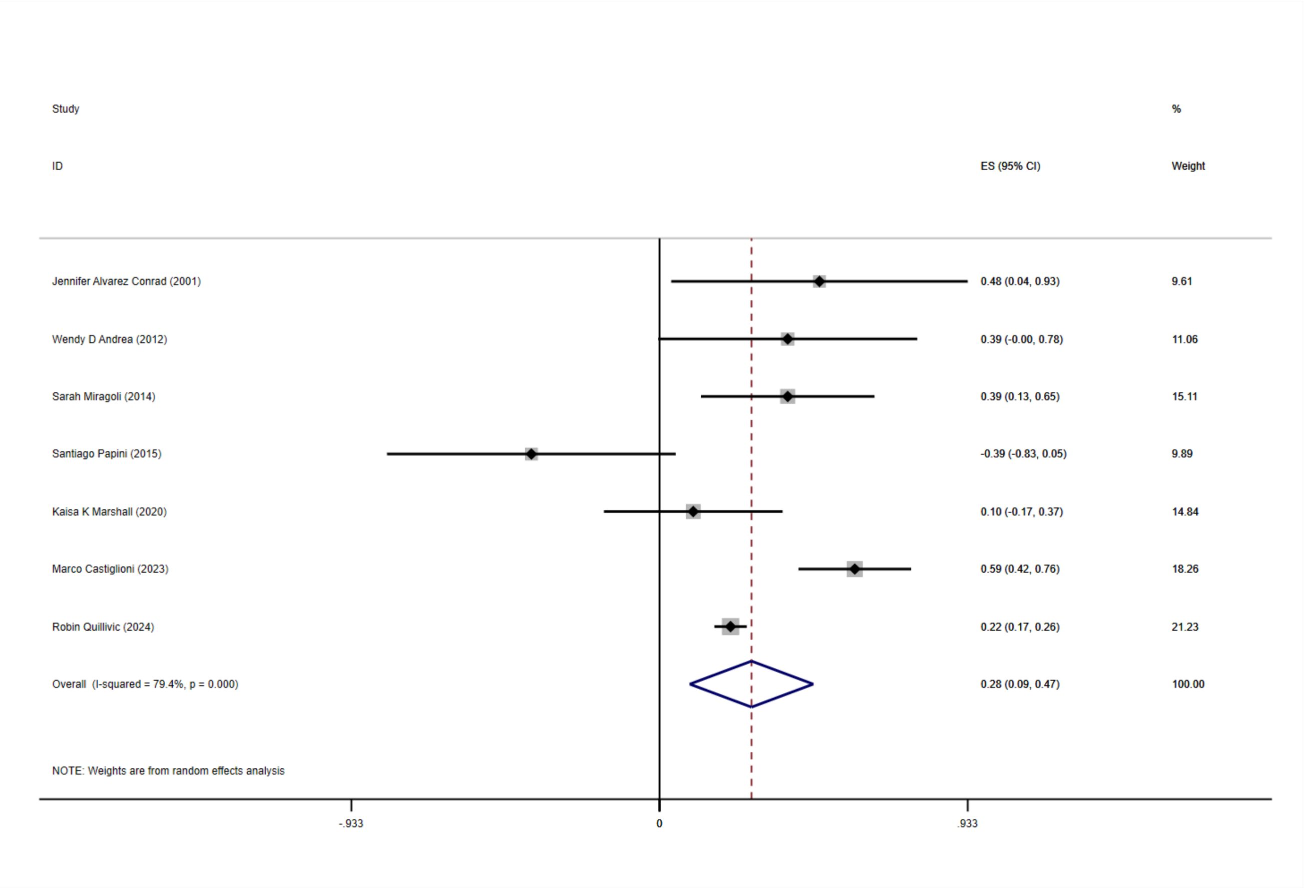

Supplementary Figure 5. death-related words and PTSD symptoms

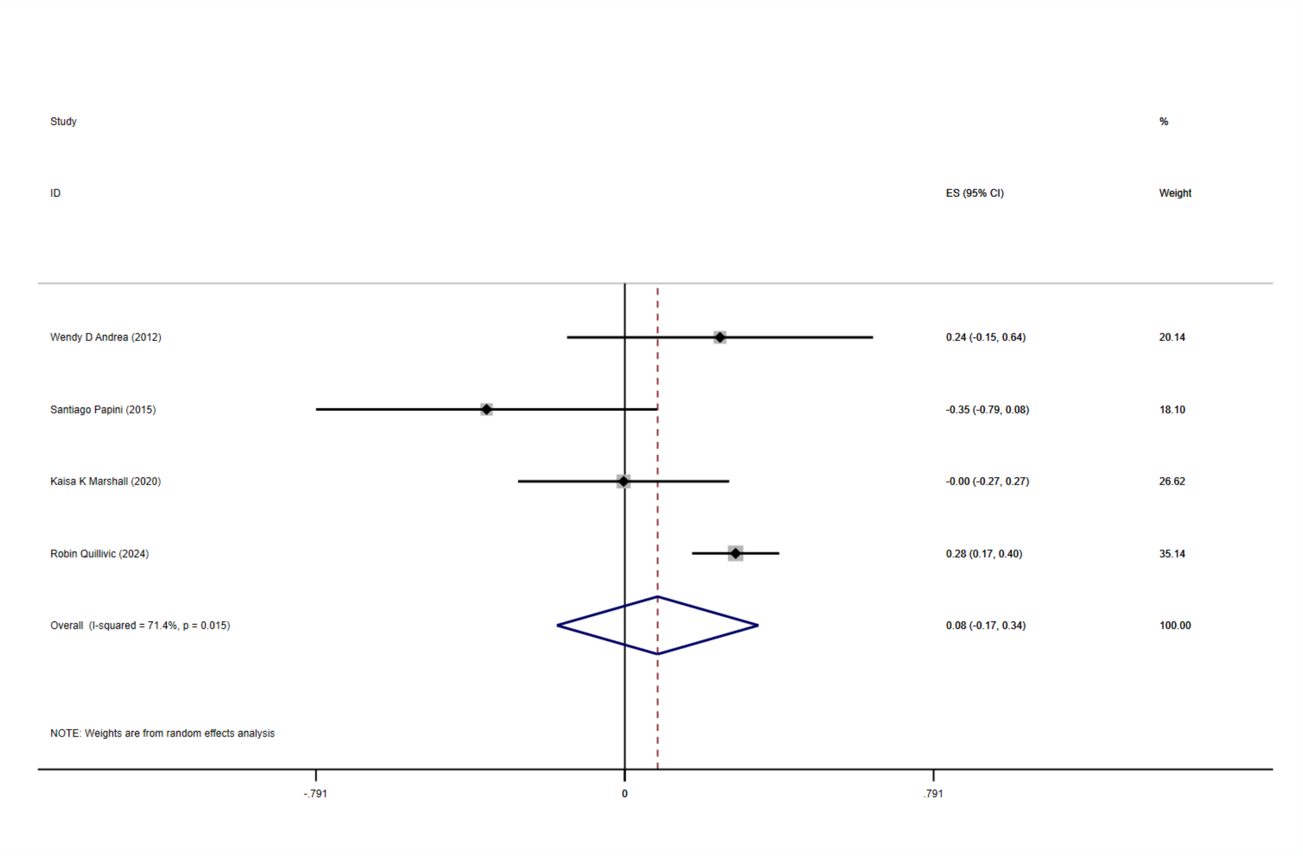

Supplementary Figure 6. death-related words and hyperarousal symptoms

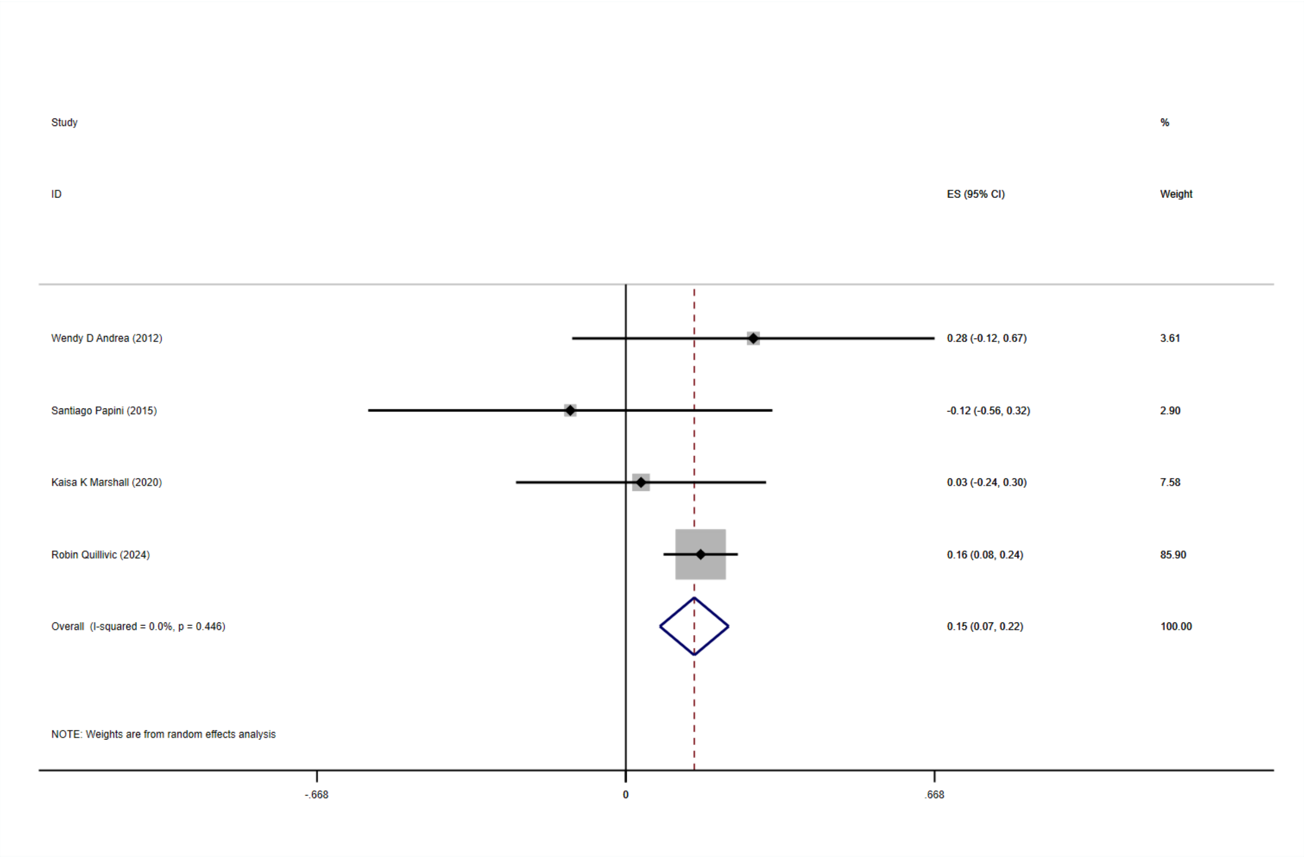

Supplementary Figure 7. death-related words and intrusive symptoms

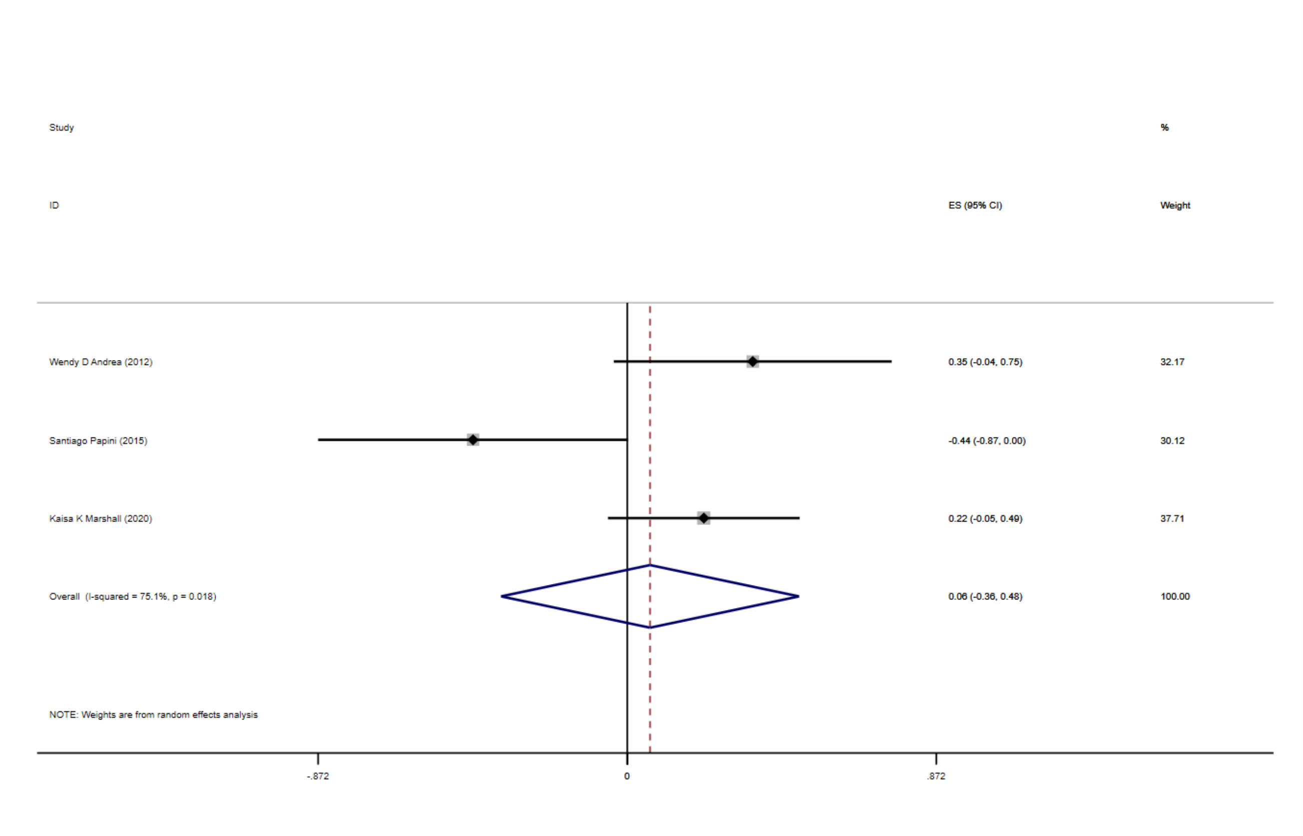

Supplementary Figure 8. death-related words and avoidance symptoms

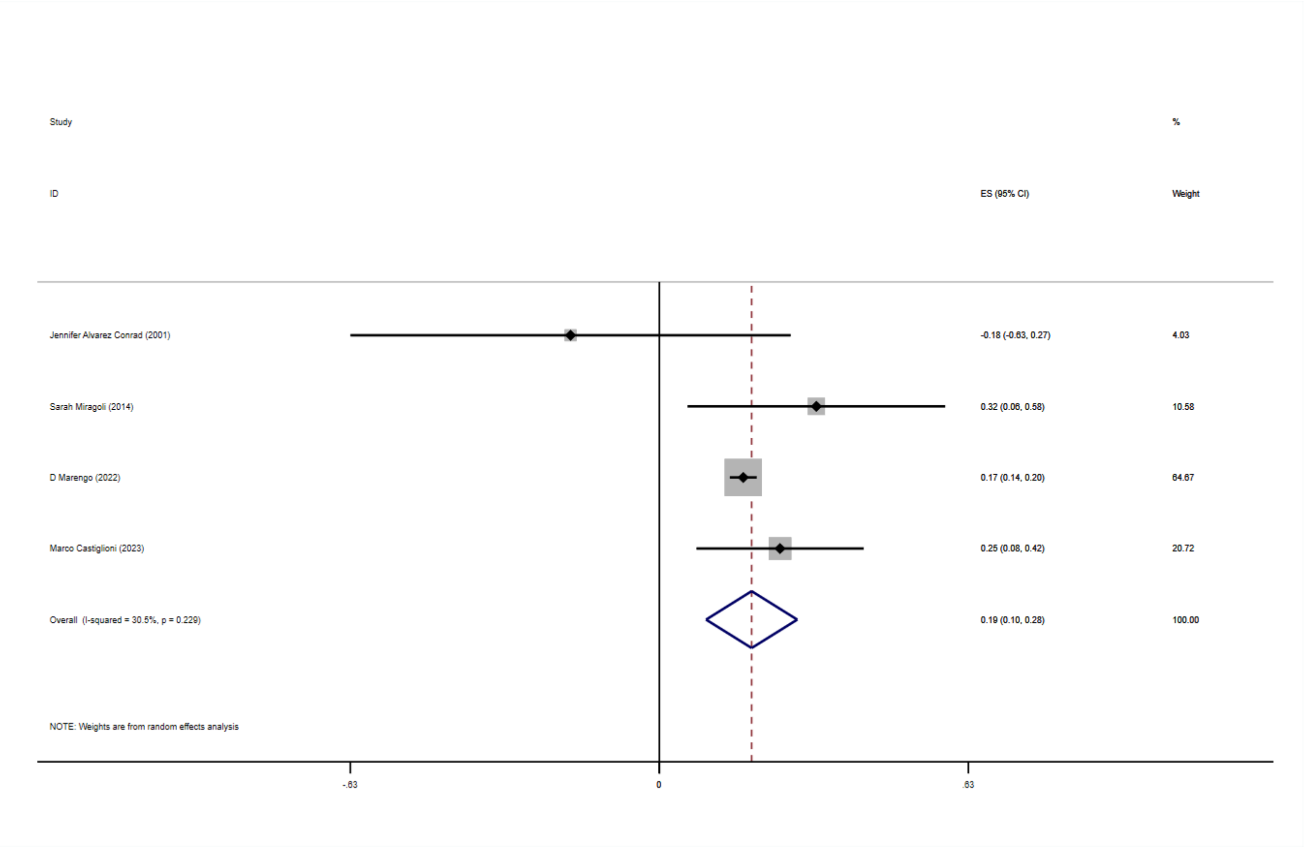

Supplementary Figure 9. negative emotion-related words and PTSD symptoms

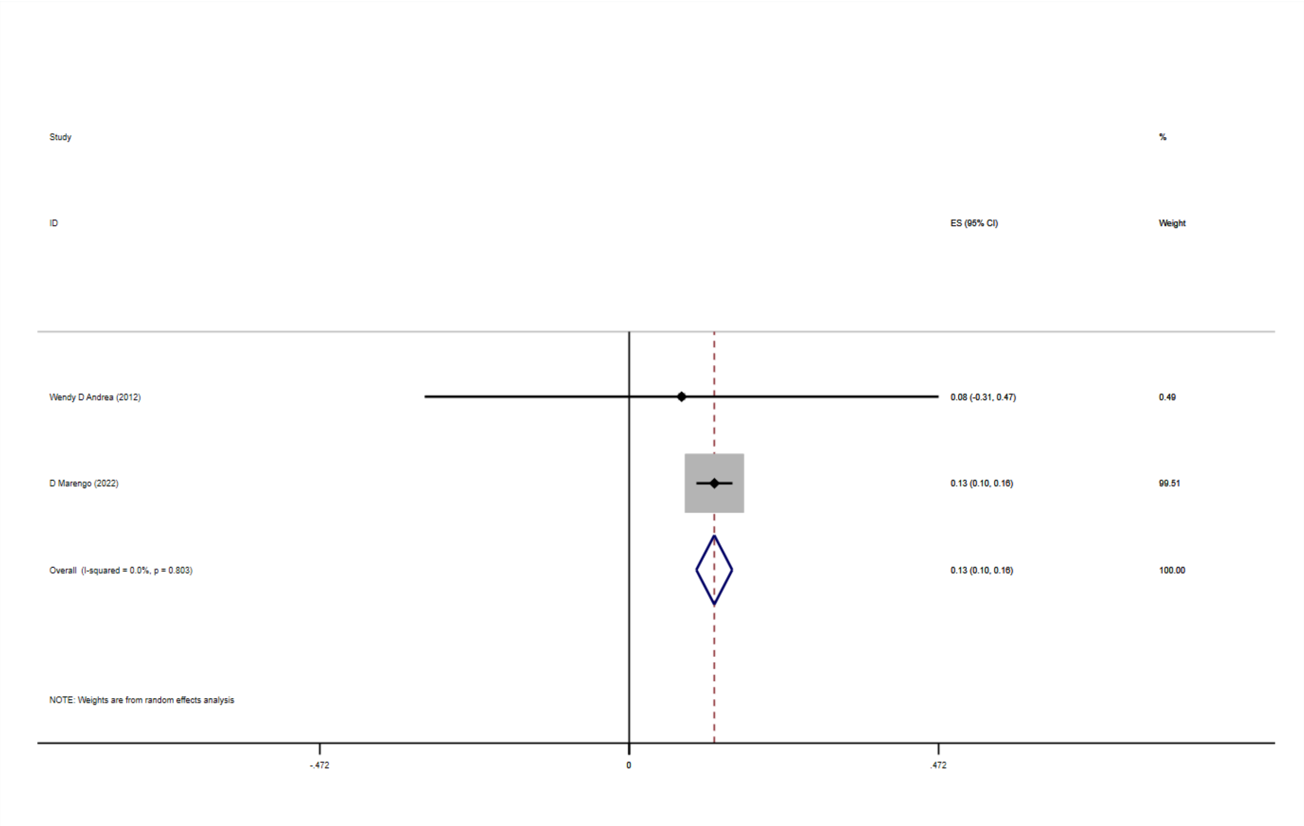

Supplementary Figure 10. anger-related words and PTSD symptoms

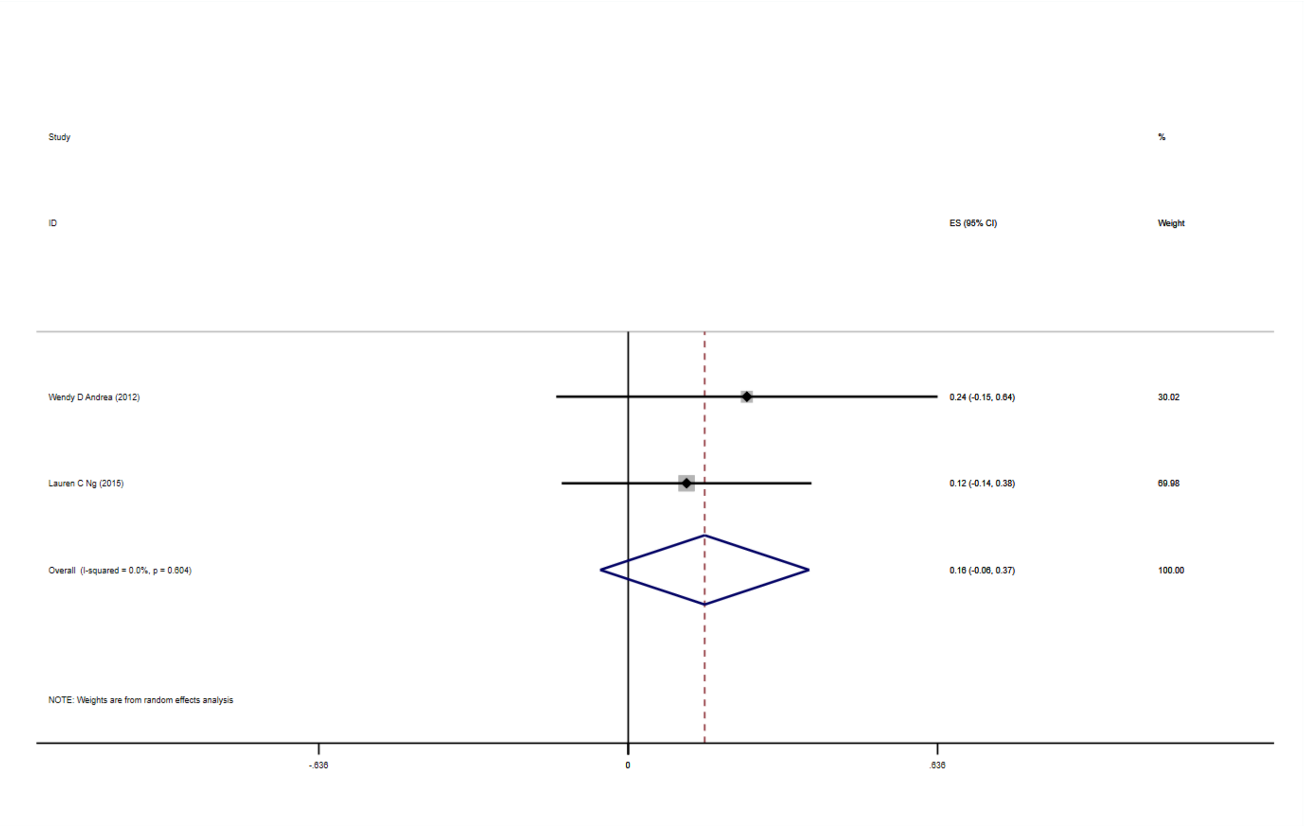

Supplementary Figure 11. anger-related words and hyperarousal symptoms

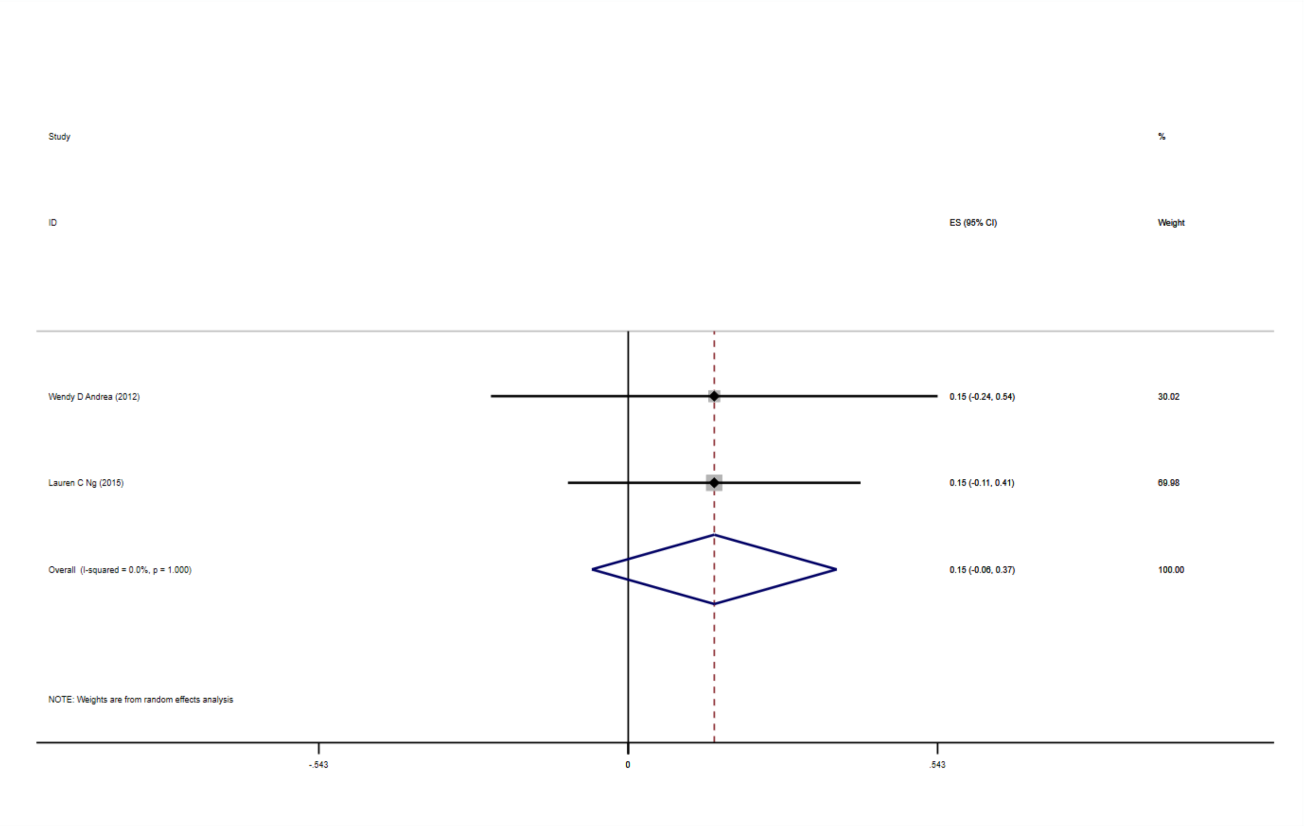

Supplementary Figure 12. anger-related words and avoidance symptoms

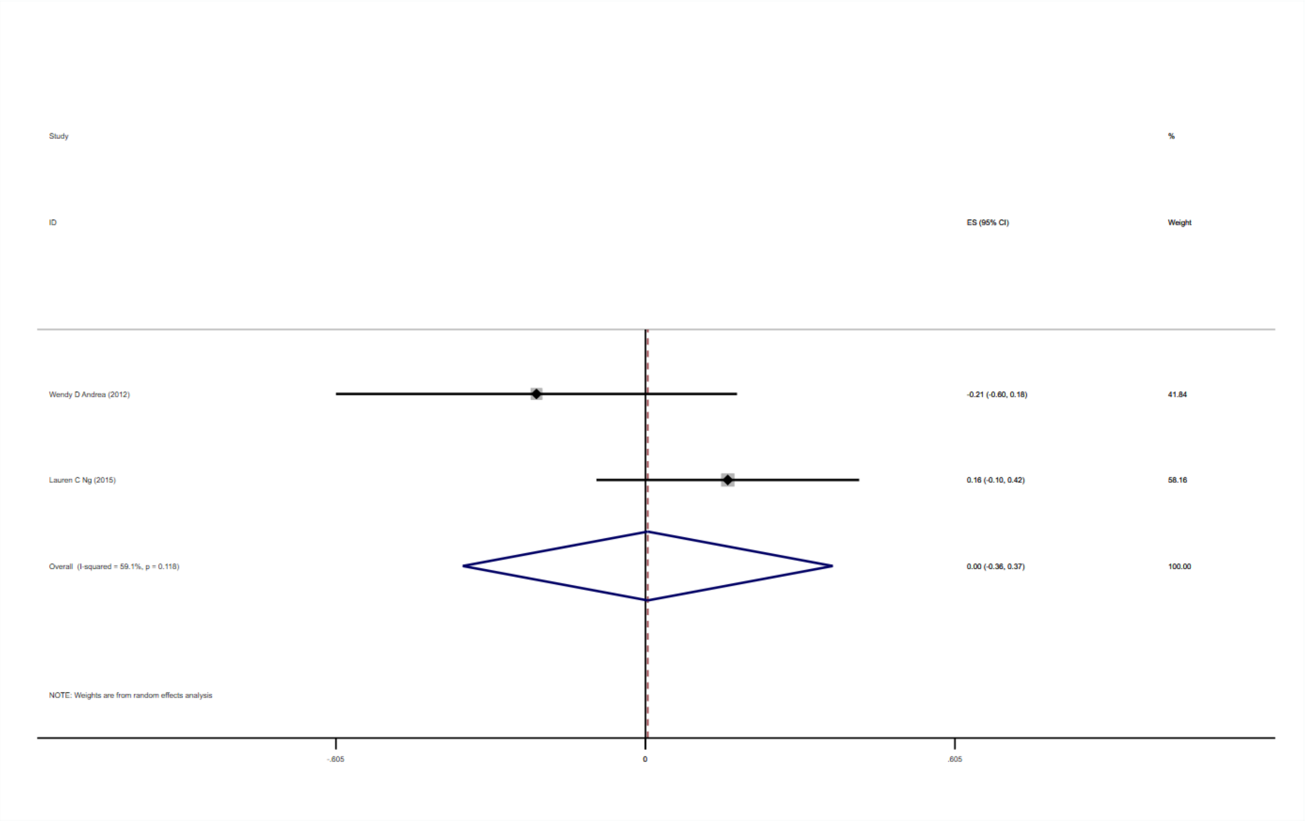

Supplementary Figure 13. anger-related words and intrusive symptoms

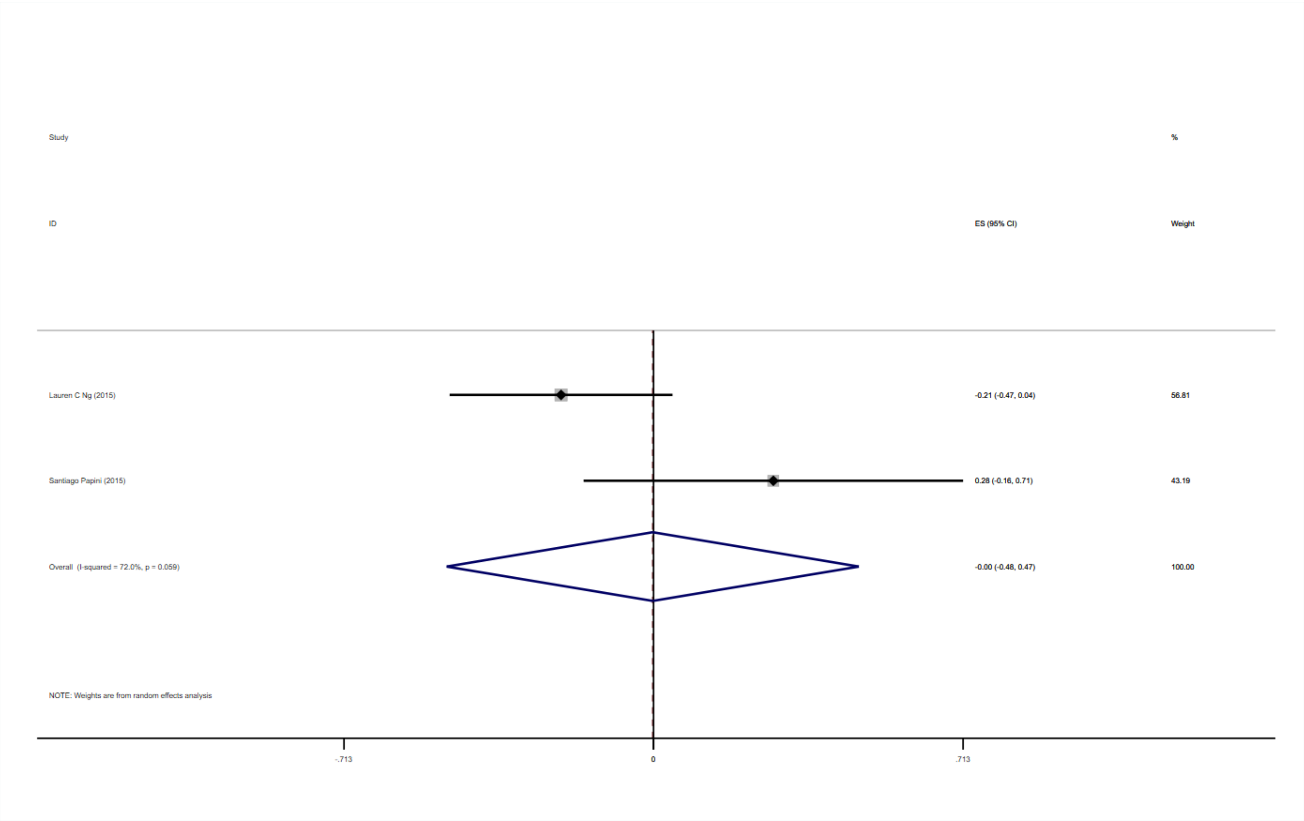

Supplementary Figure 24. sadness-related words and hyperarousal symptoms

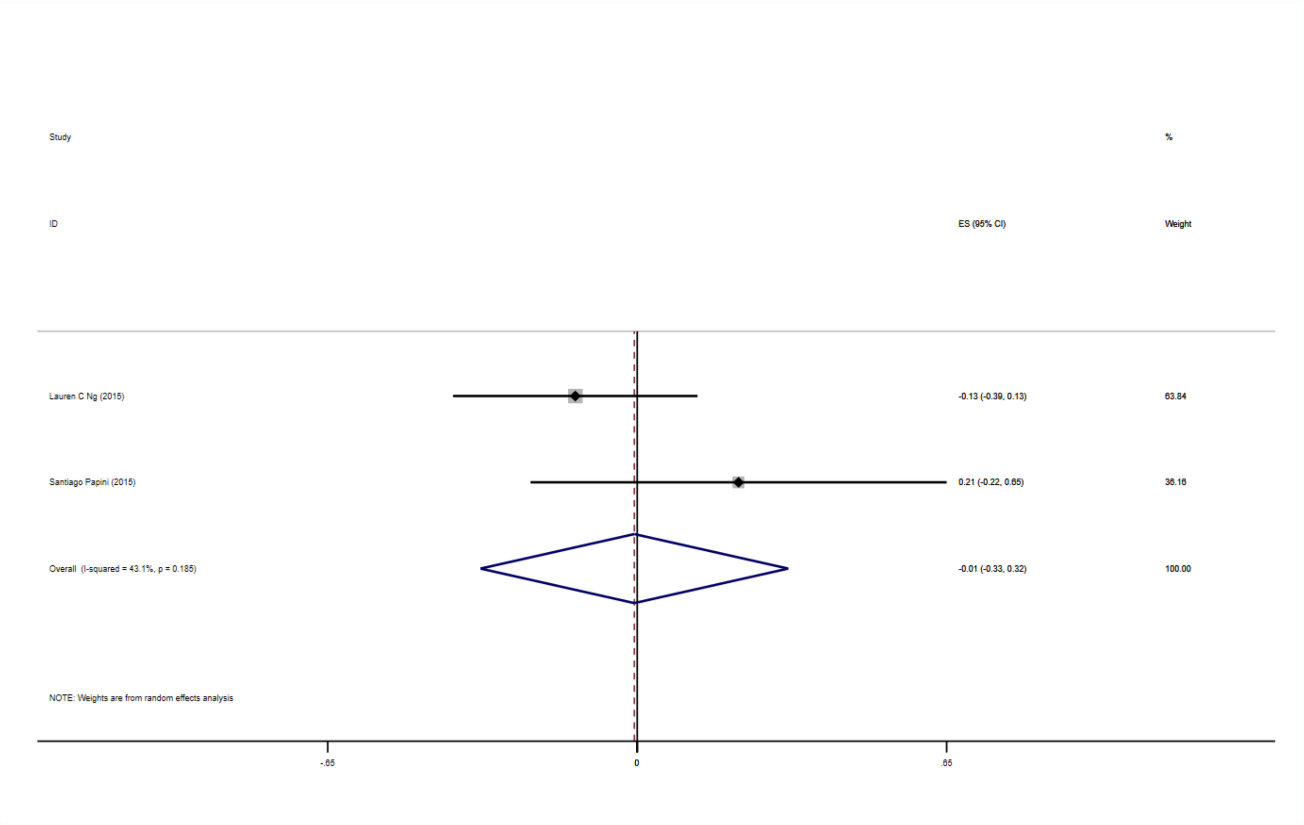

Supplementary Figure 35. sadness-related words and avoidance symptoms

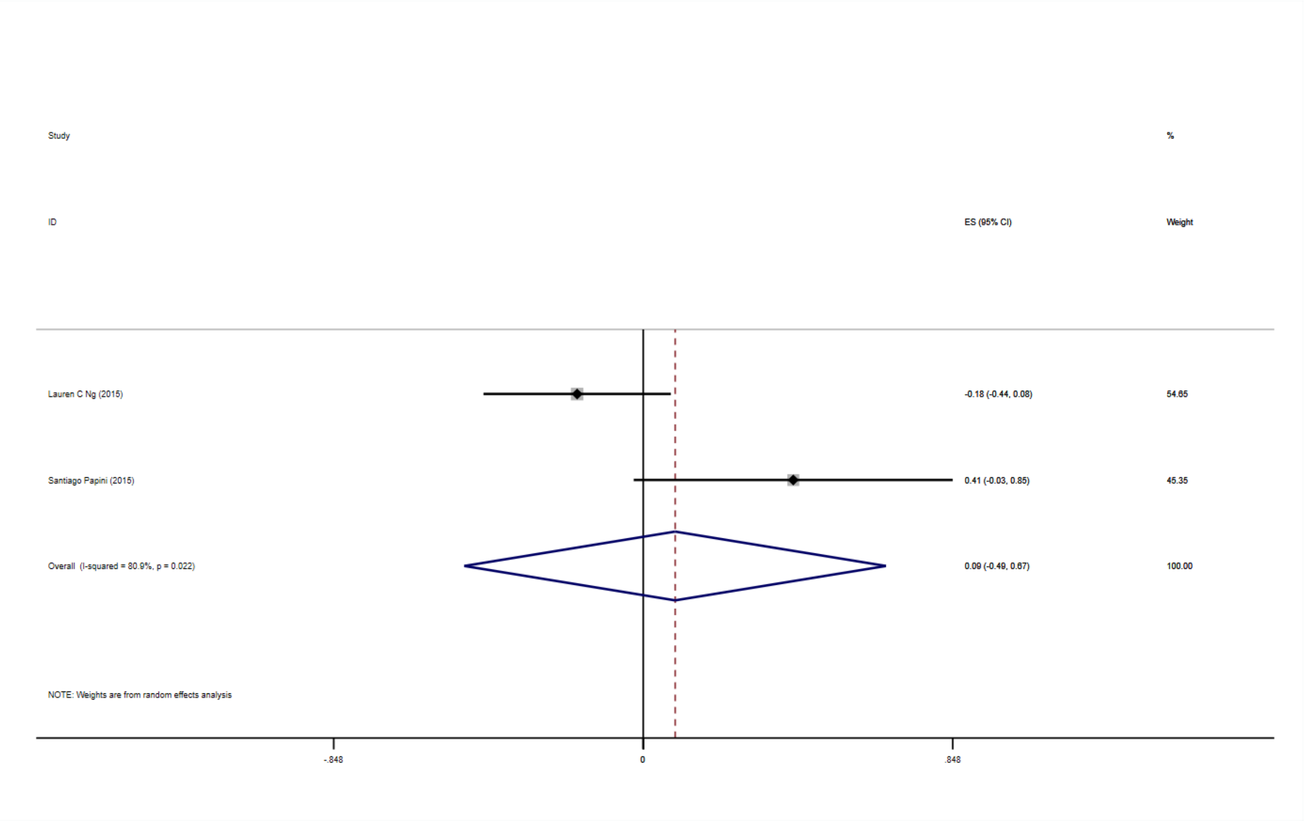

Supplementary Figure 46. sadness-related words and intrusive symptoms

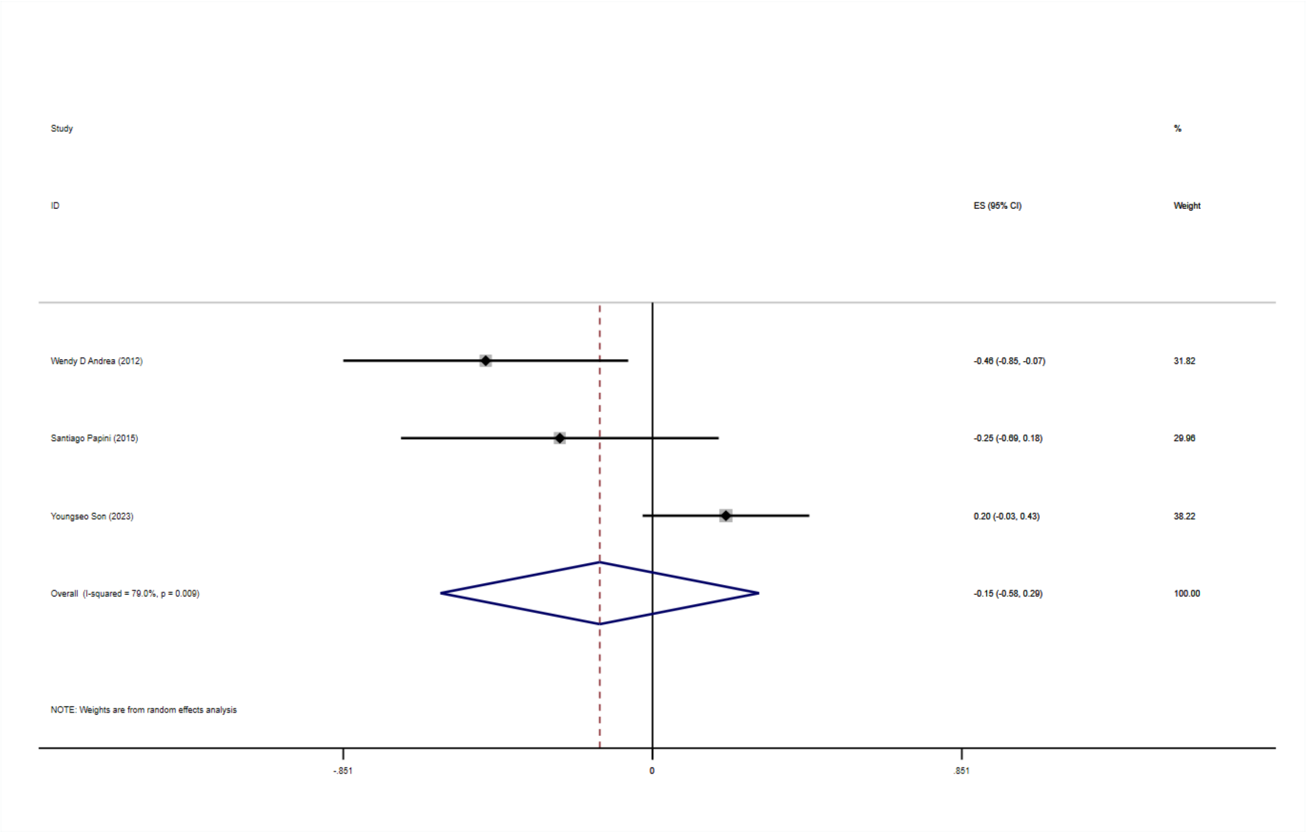

Supplementary Figure 17. anxiety-related words and PTSD symptoms

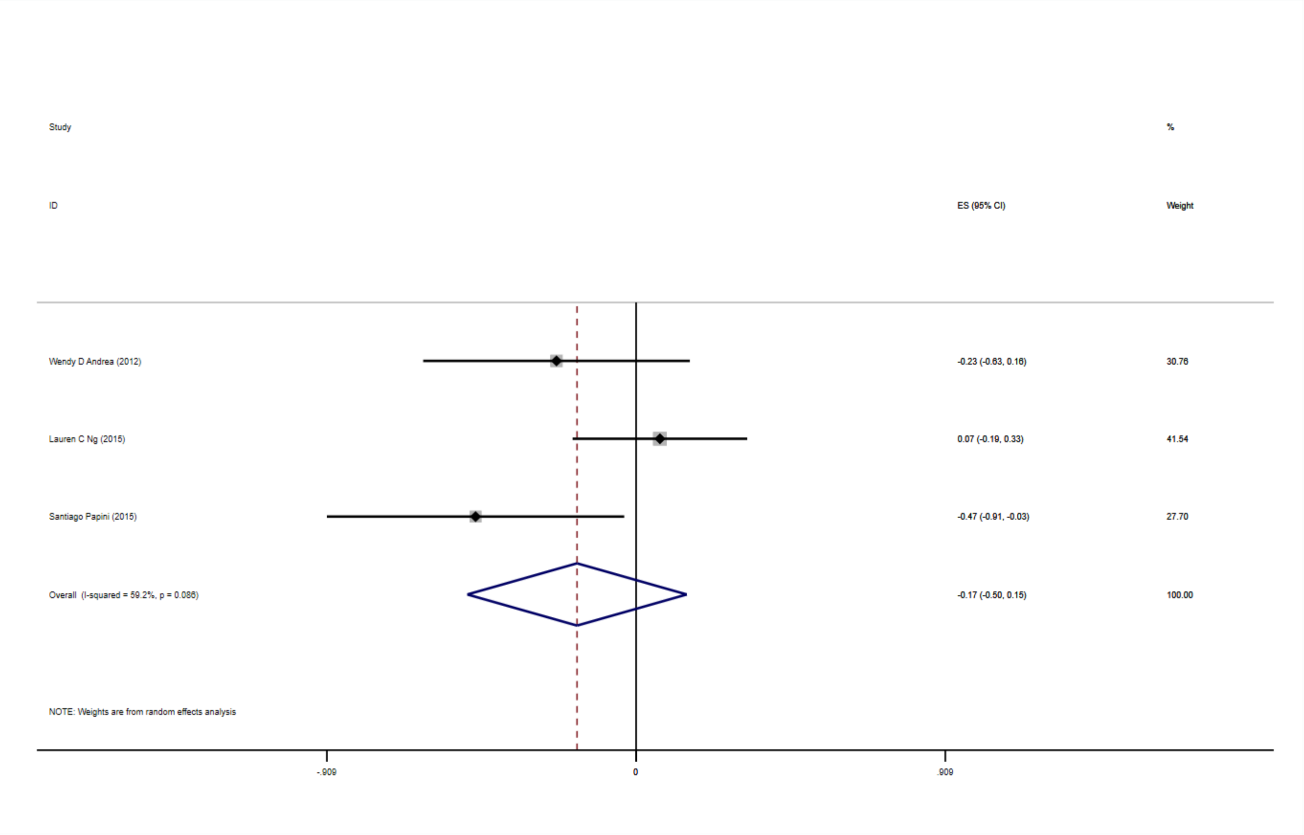

Supplementary Figure 58. anxiety-related words and hyperarousal symptoms

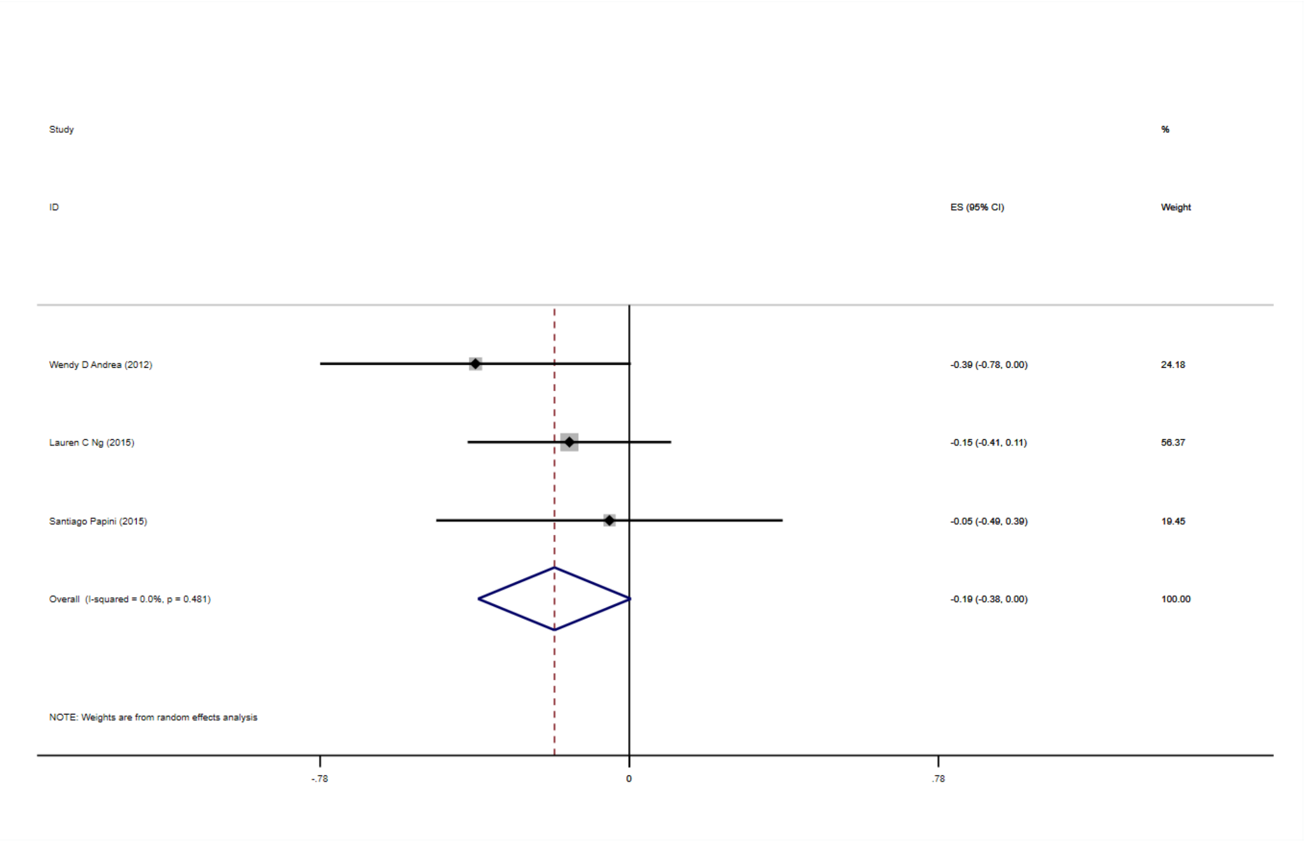

Supplementary Figure 19. anxiety-related words and avoidance symptoms

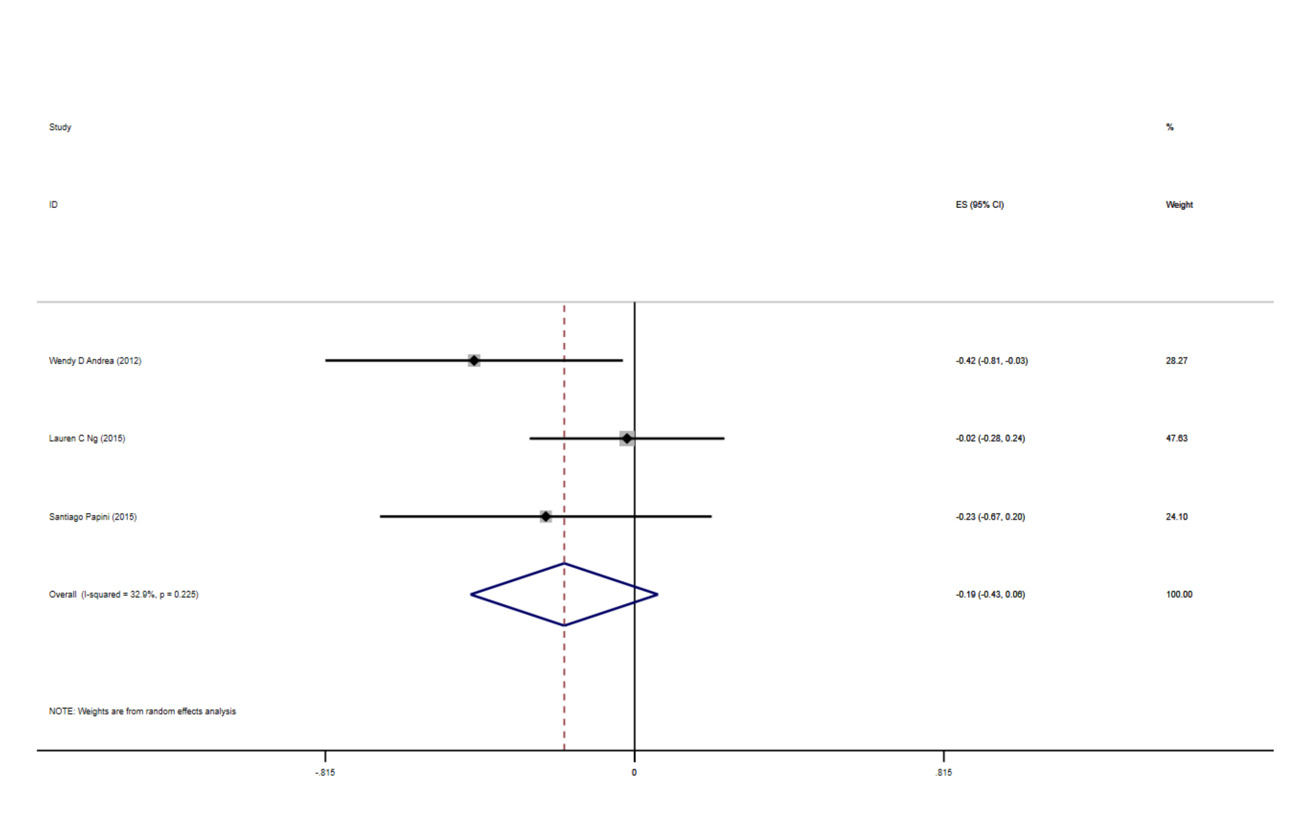

Supplementary Figure 20. anxiety-related words and intrusive symptoms

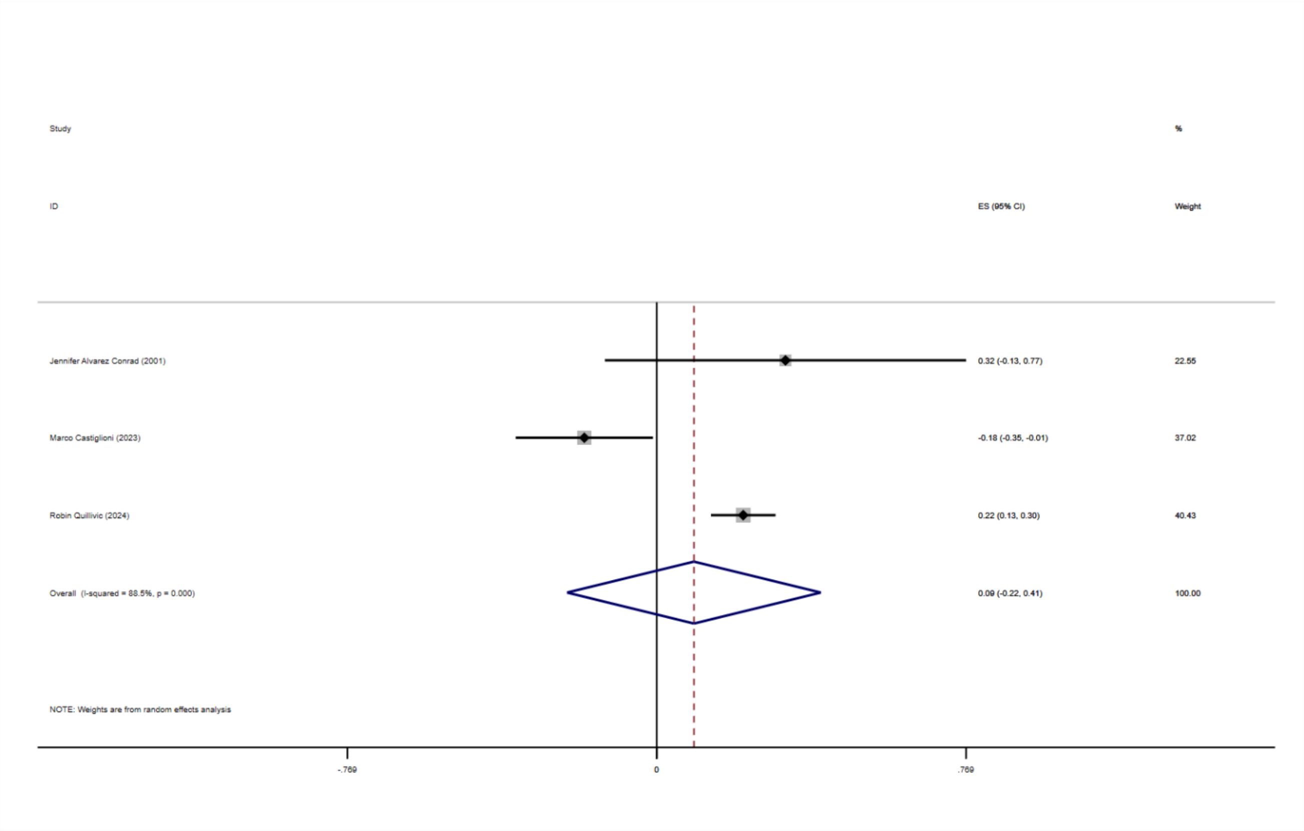

Supplementary Figure 26. positive emotion-related words and PTSD symptoms

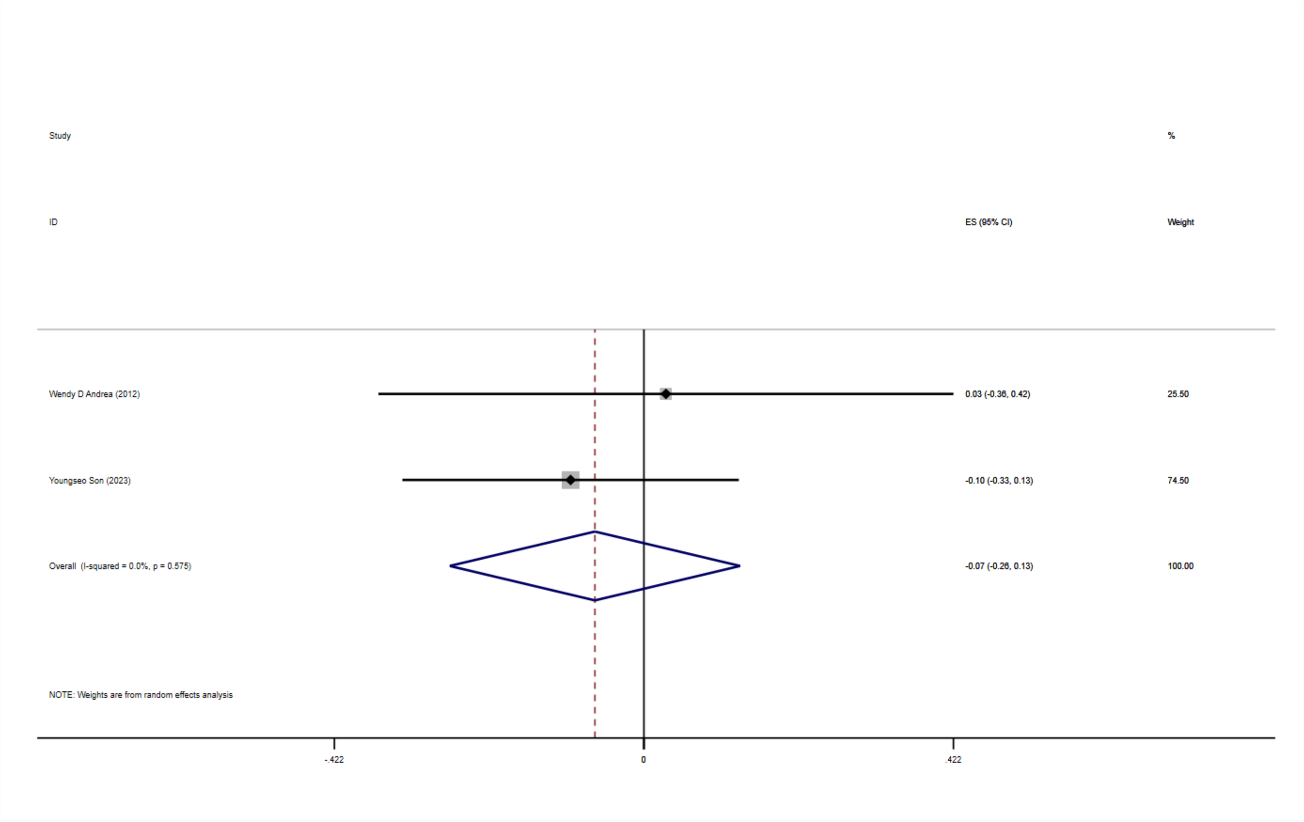

Supplementary Figure 22. first-person singular pronouns and PTSD symptoms

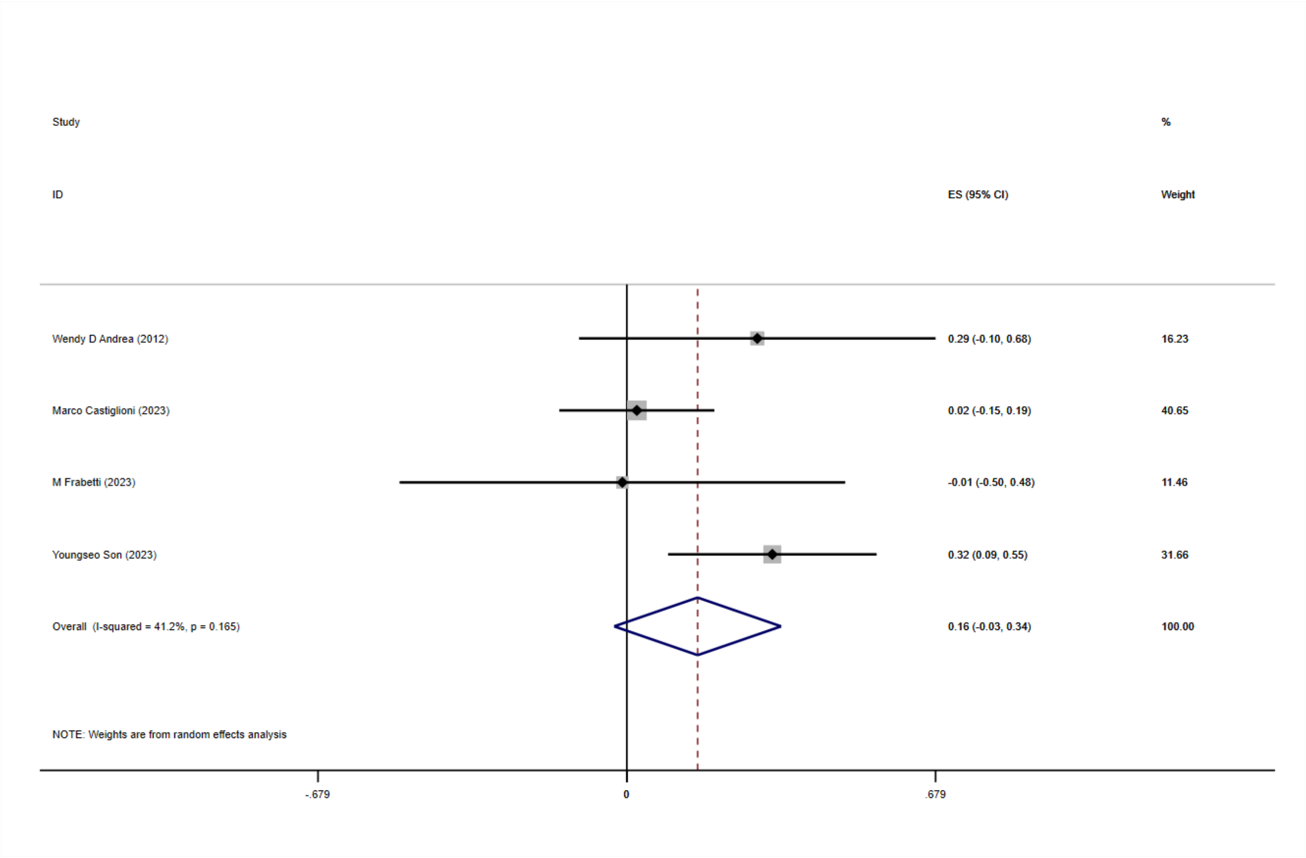

Supplementary Figure 23. first-person plural pronouns and PTSD symptoms

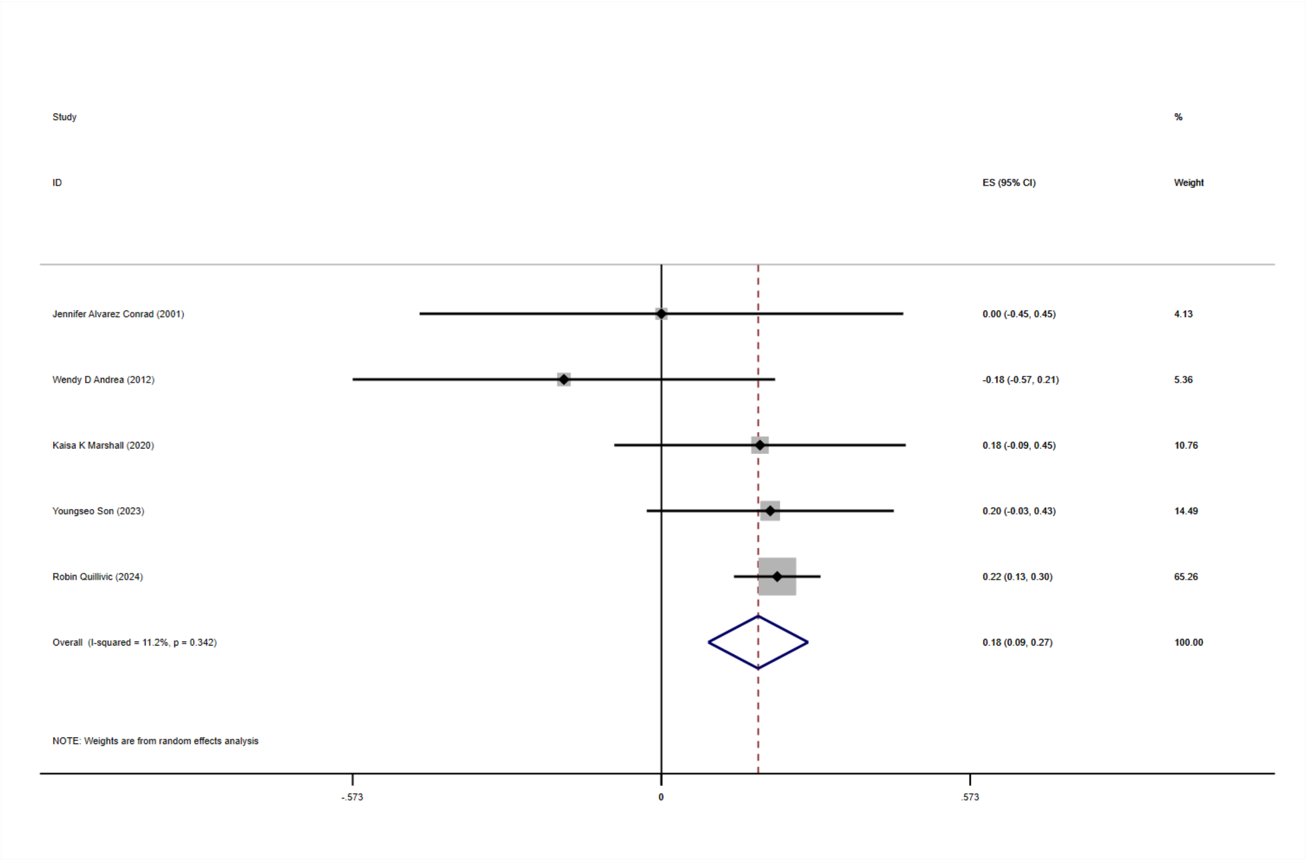

Supplementary Figure 24. word count and PTSD symptoms

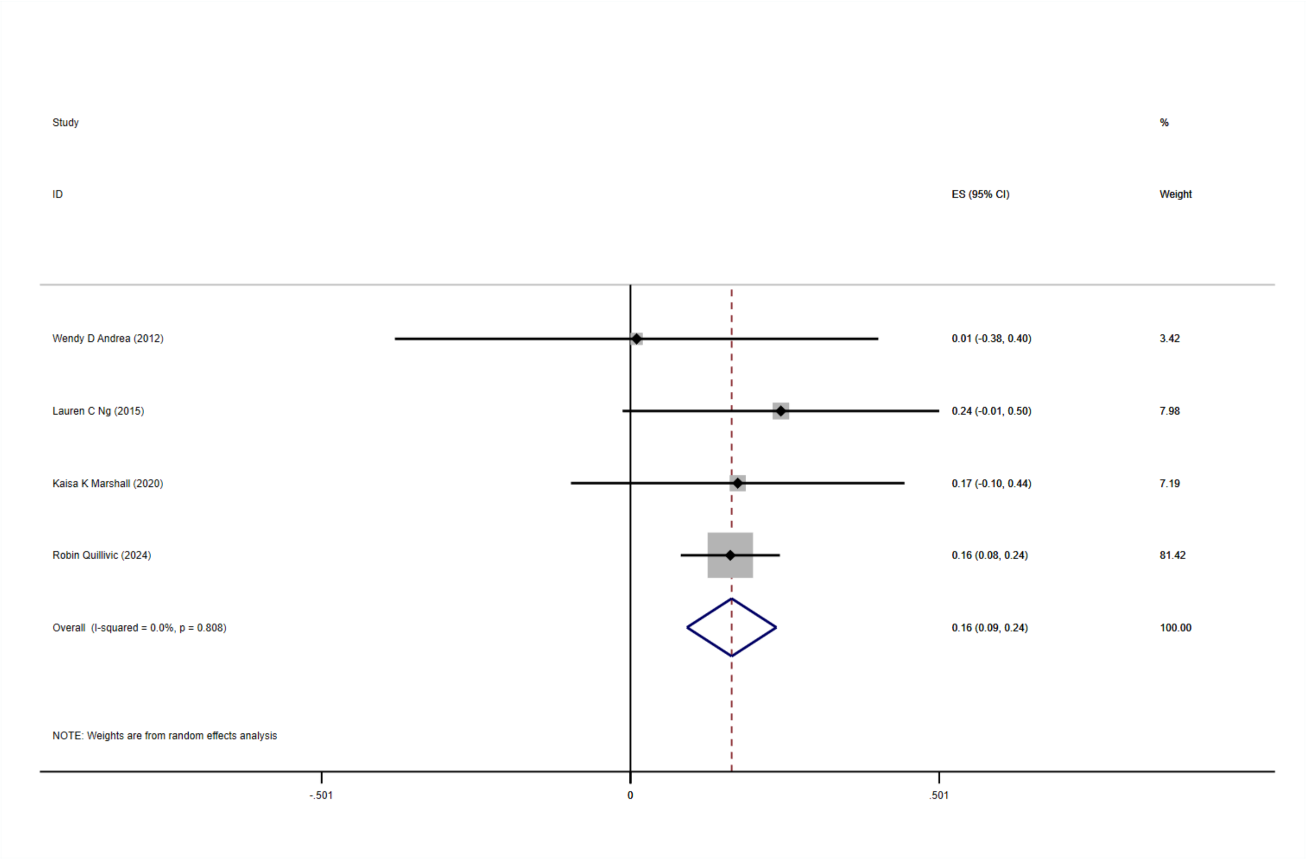

Supplementary Figure 25. word count and intrusive symptoms

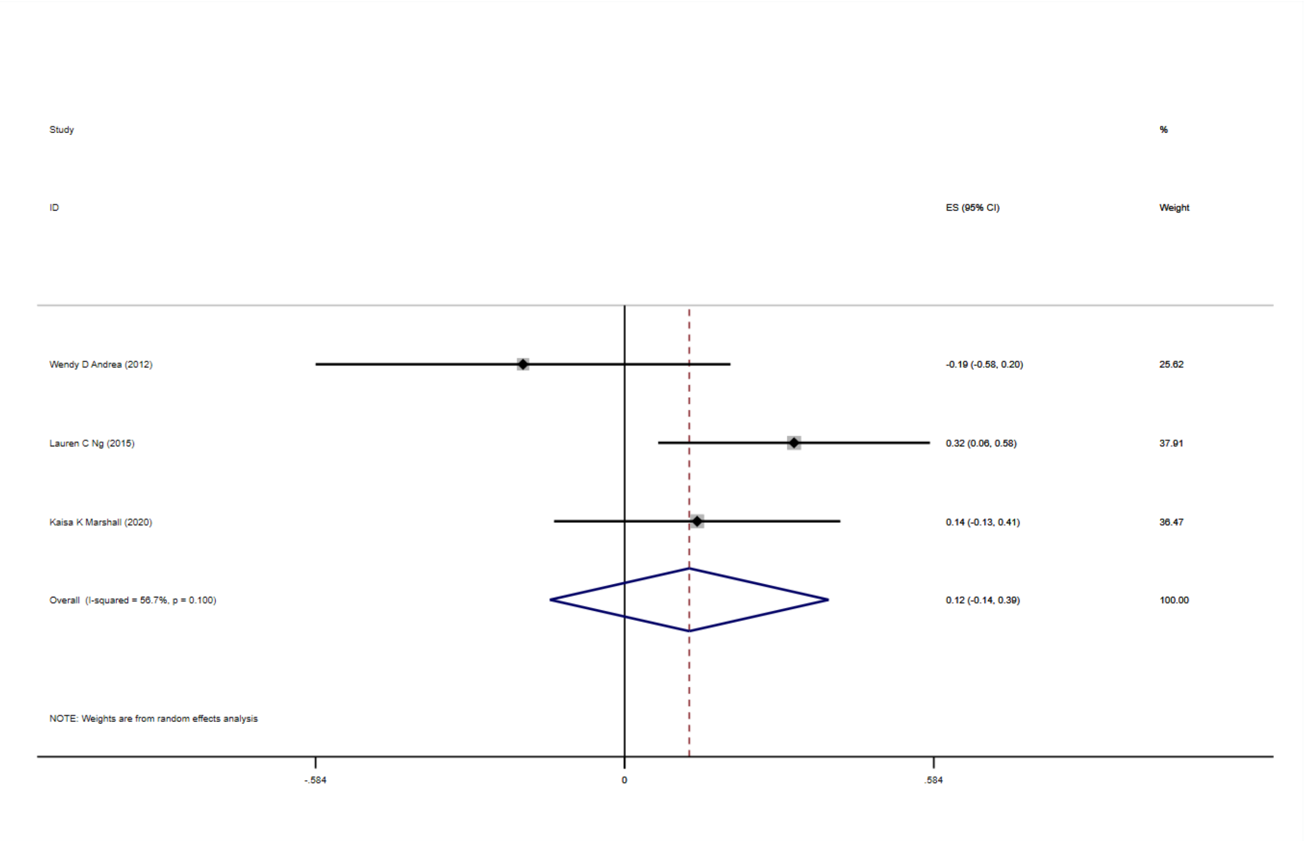

Supplementary Figure 26. word count and hyperarousal symptoms

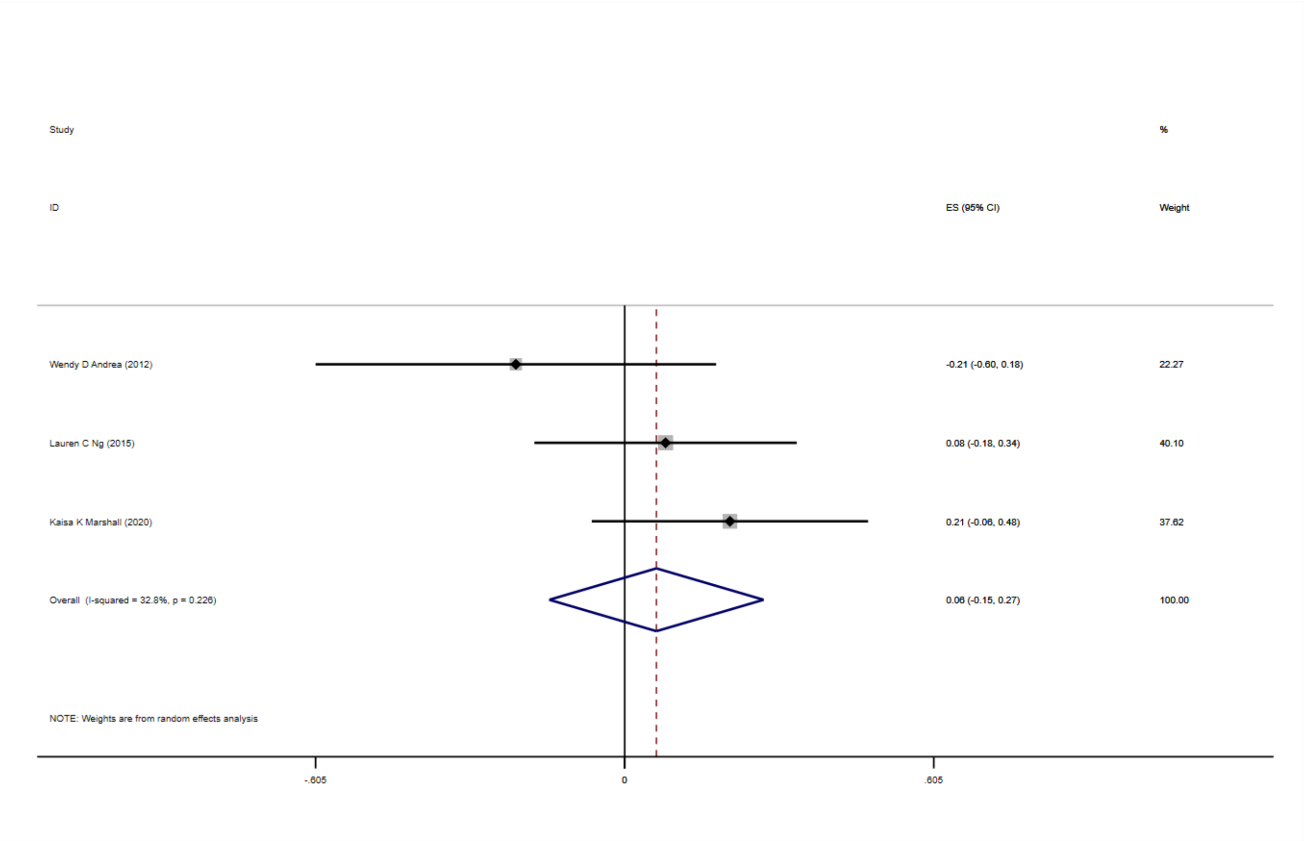

Supplementary Figure 27. word count and avoidance symptoms

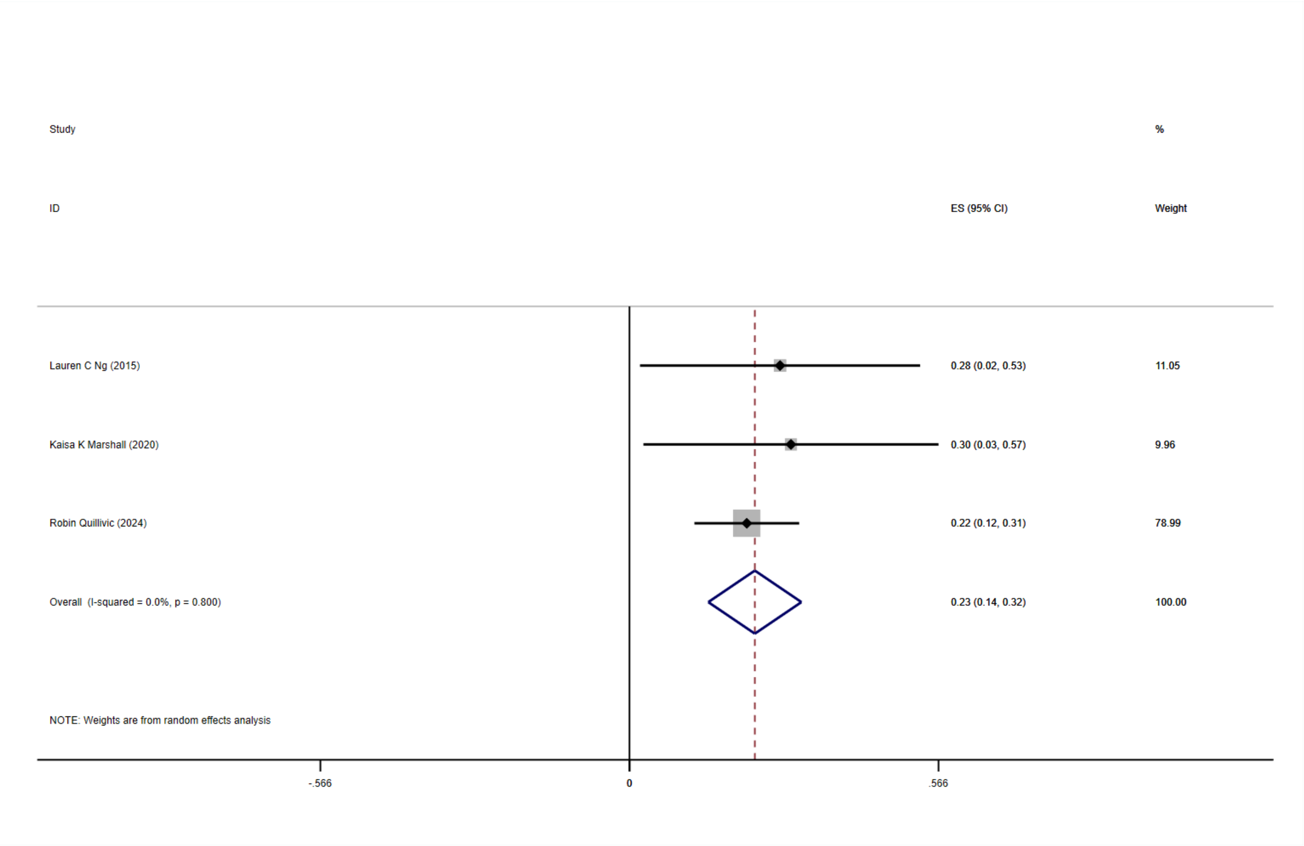

Supplementary Figure 28. body-related words and hyperarousal symptoms

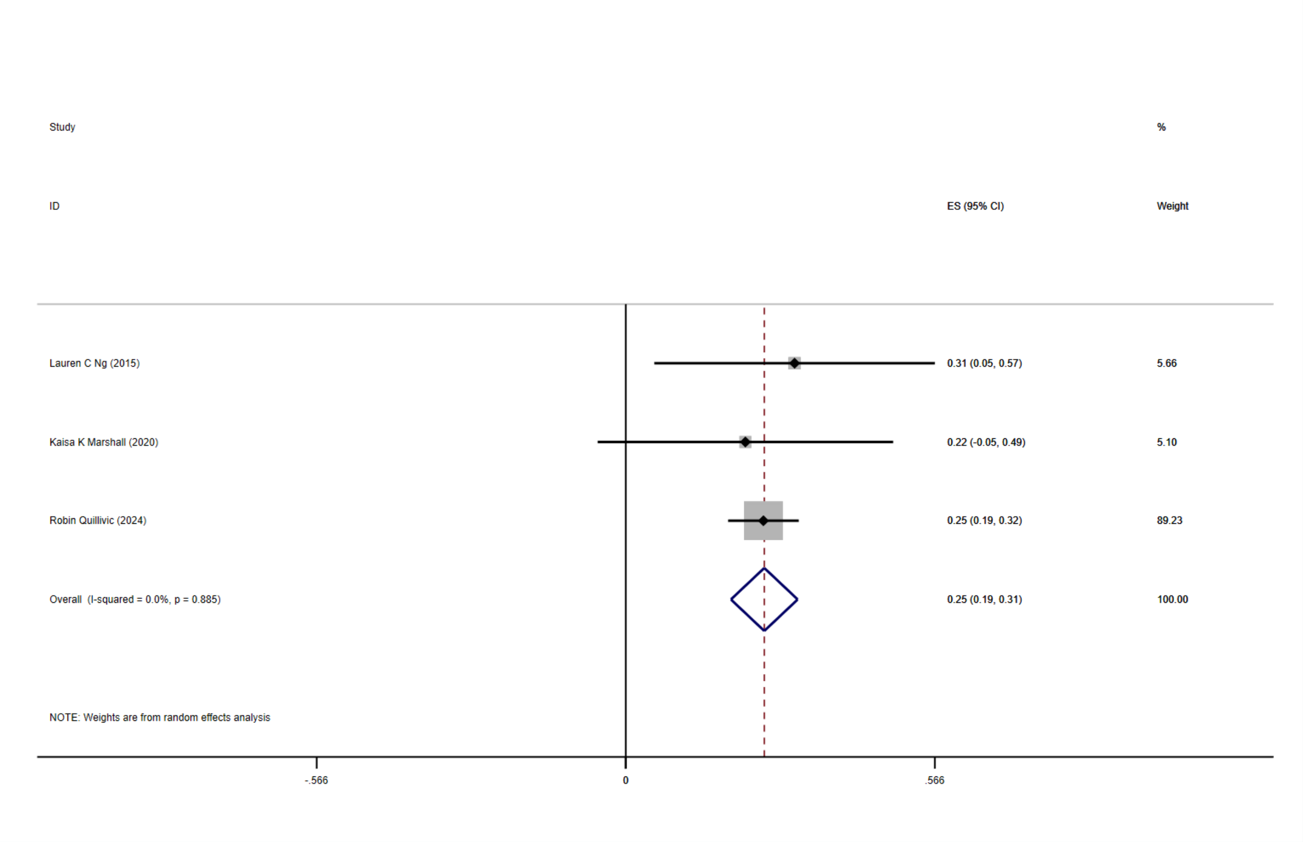

Supplementary Figure 29. body-related words and avoidance symptoms

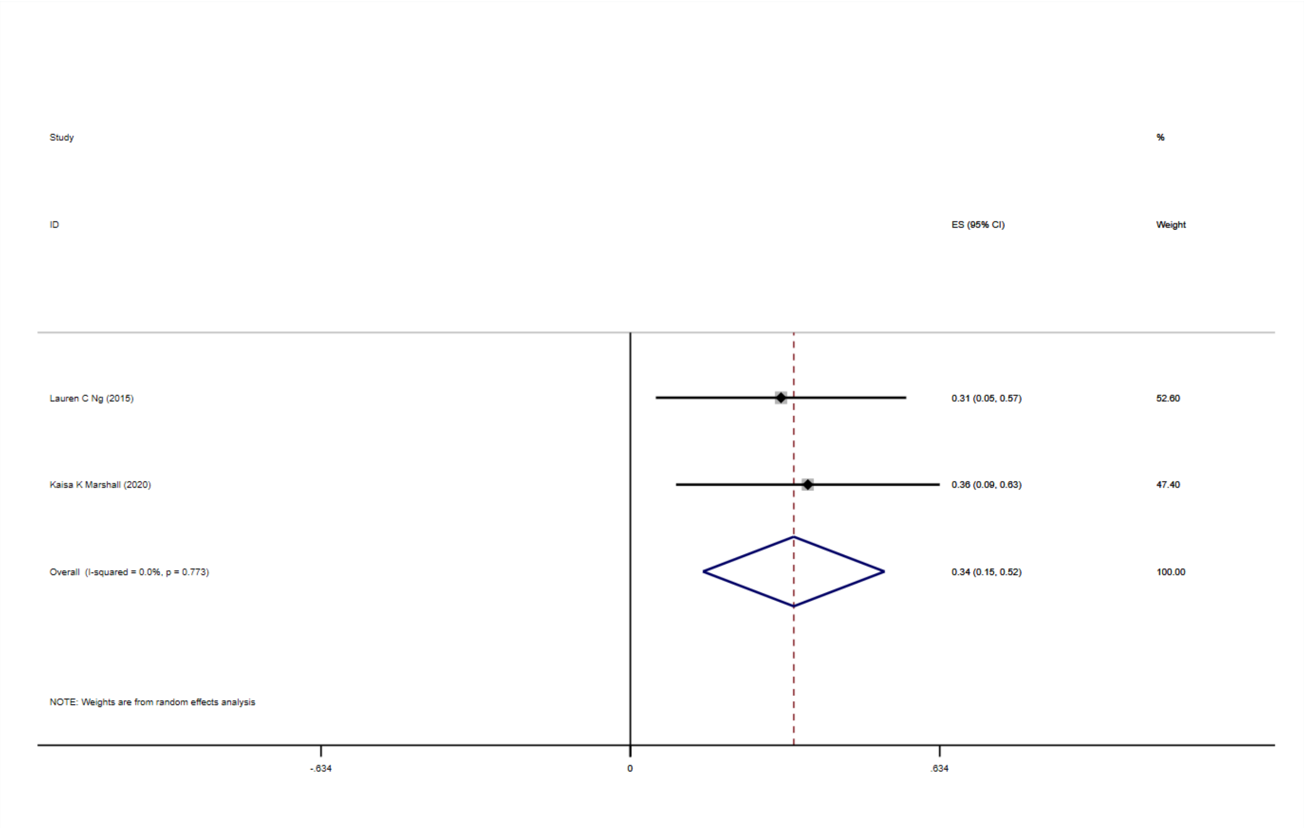

Supplementary Figure 30. body-related words and intrusive symptoms

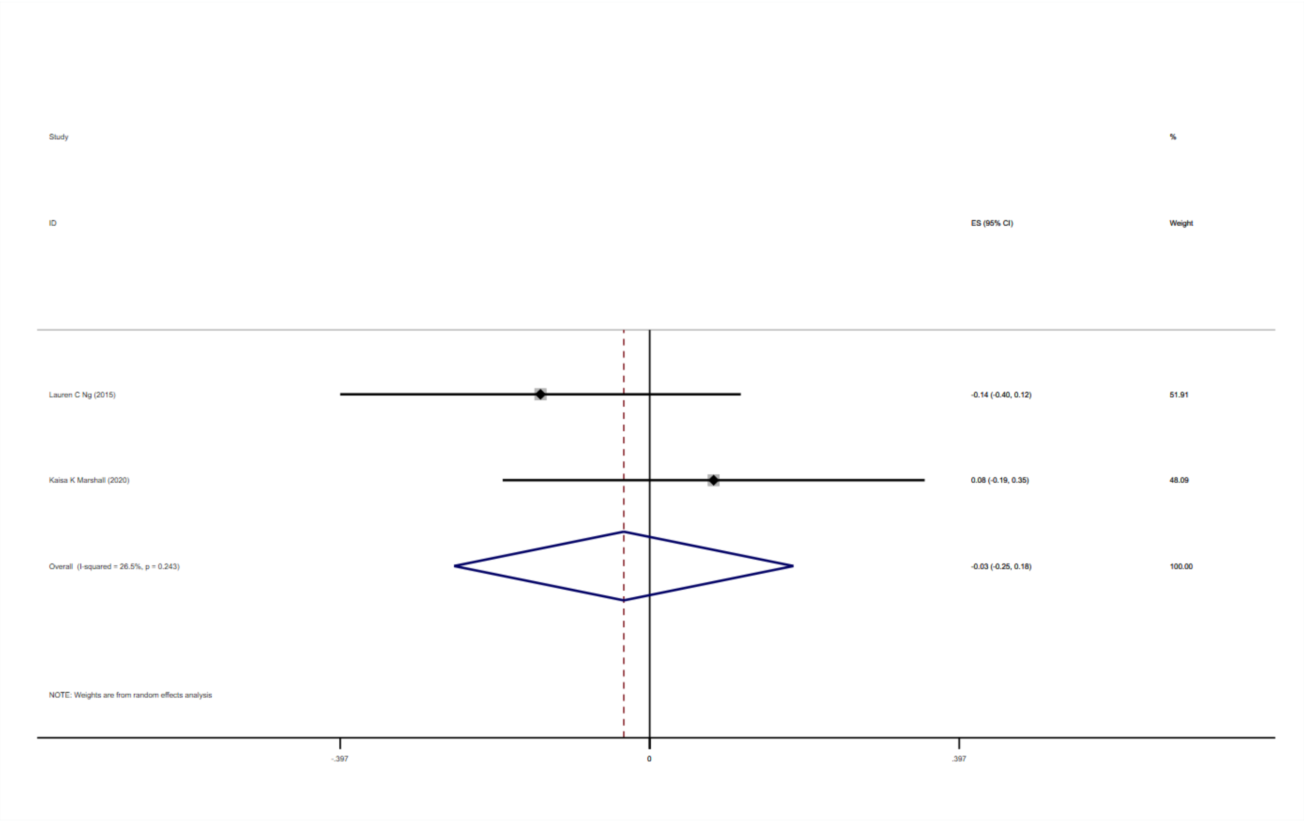

Supplementary Figure 31. seeing-related words and hyperarousal symptoms

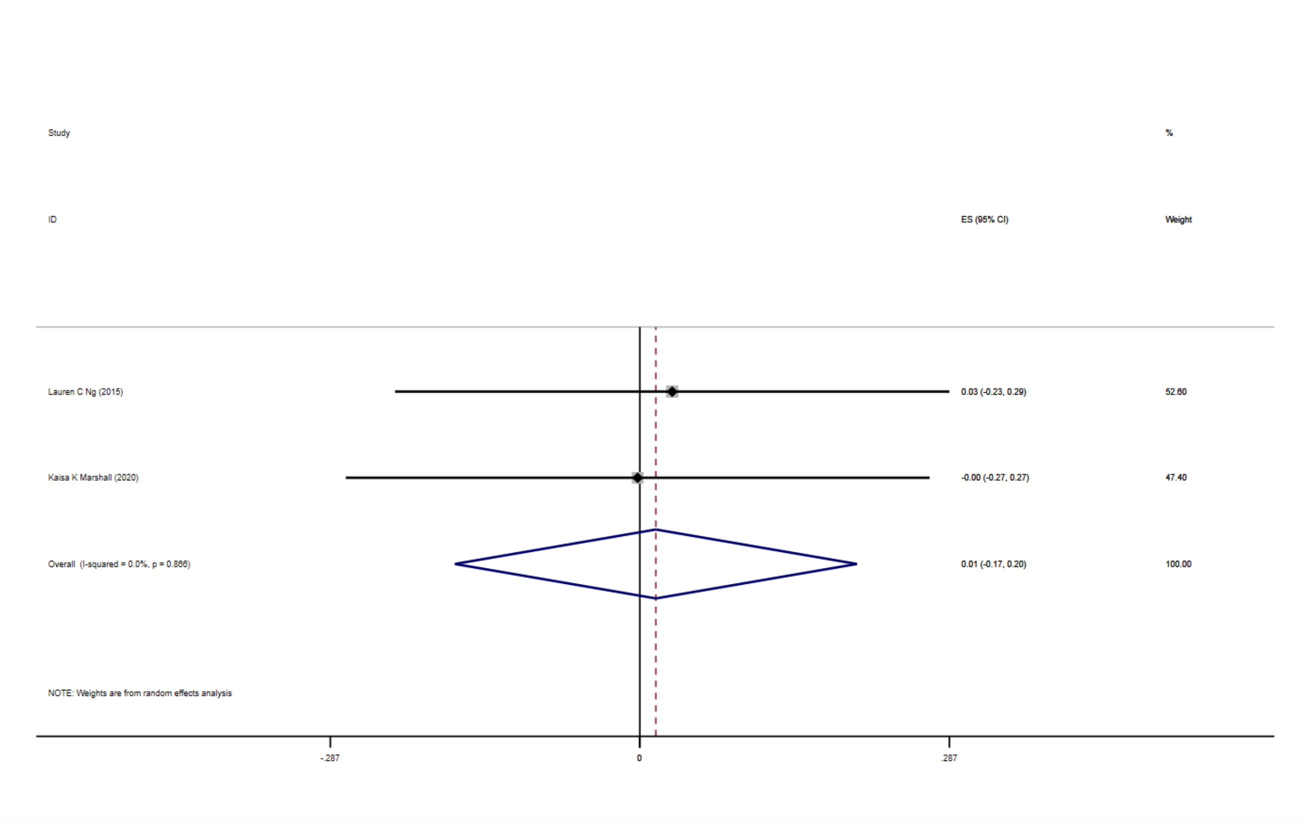

Supplementary Figure 32. seeing-related words and avoidance symptoms

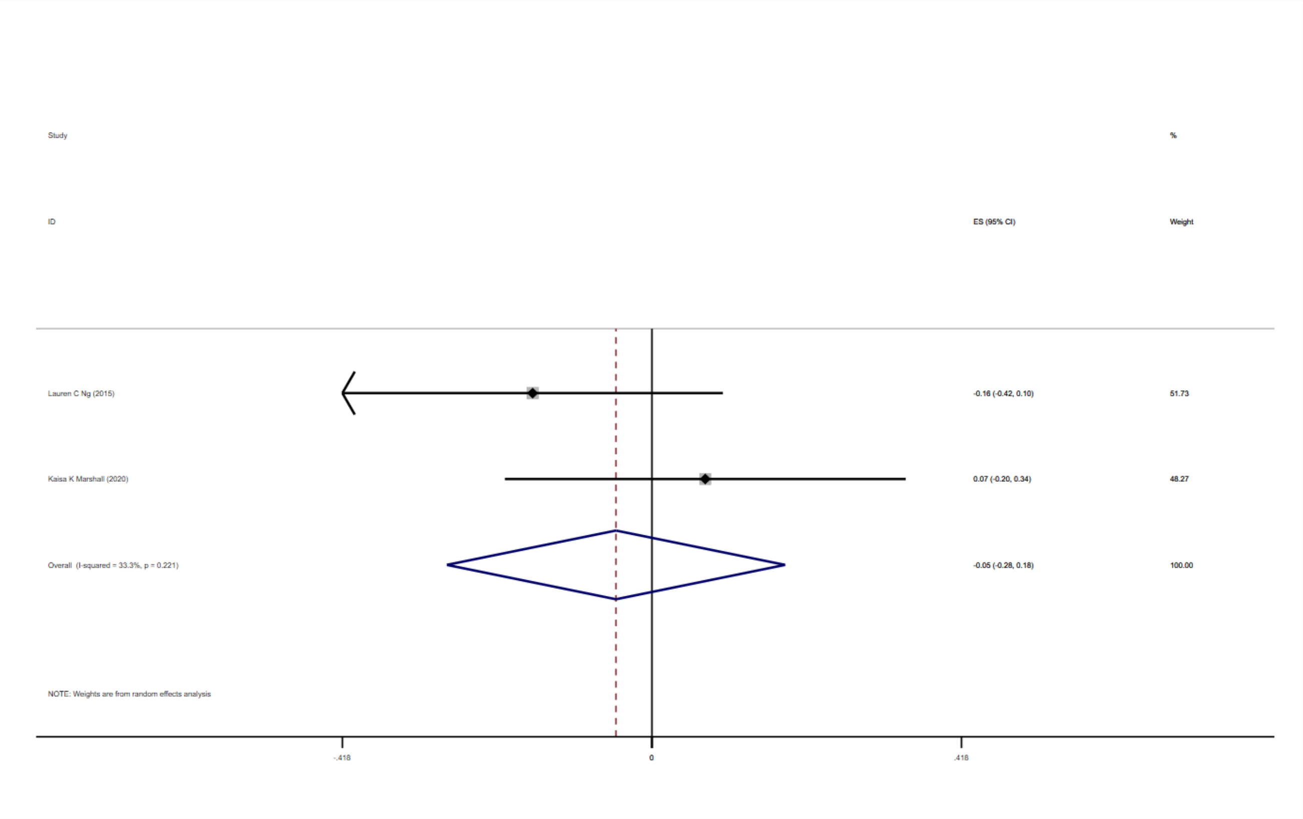

Supplementary Figure 33. seeing-related words and intrusive symptoms

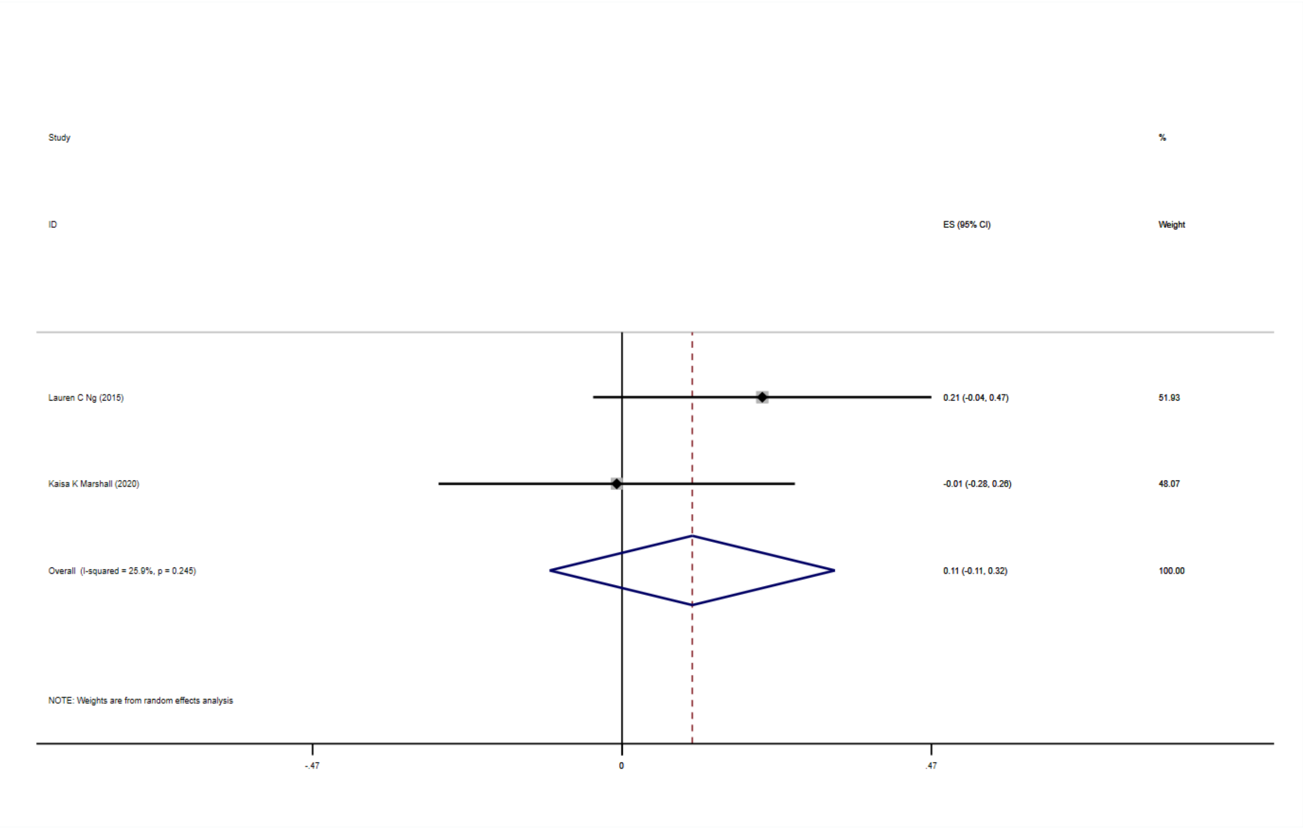

Supplementary Figure 34. touching-related words and hyperarousal symptoms

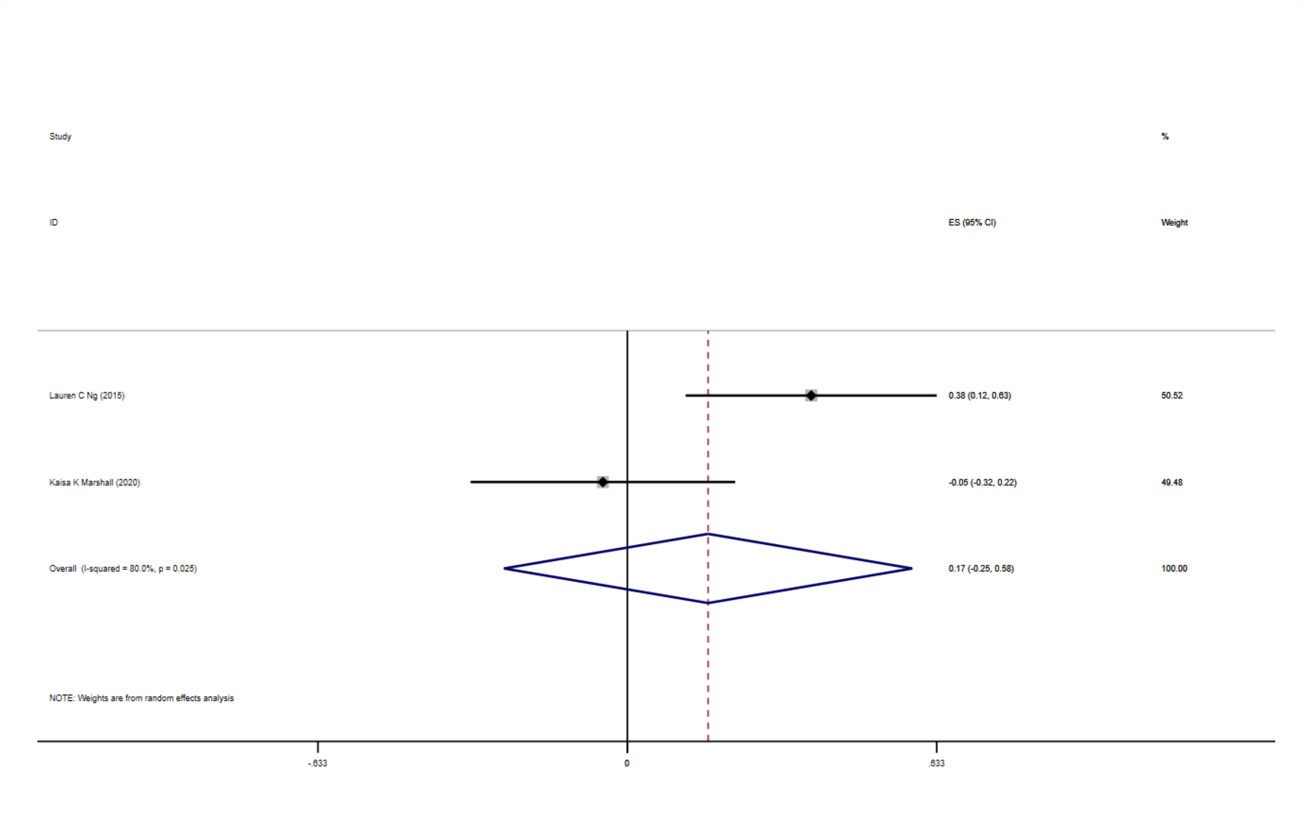

Supplementary Figure 35. touching-related words and avoidance symptoms

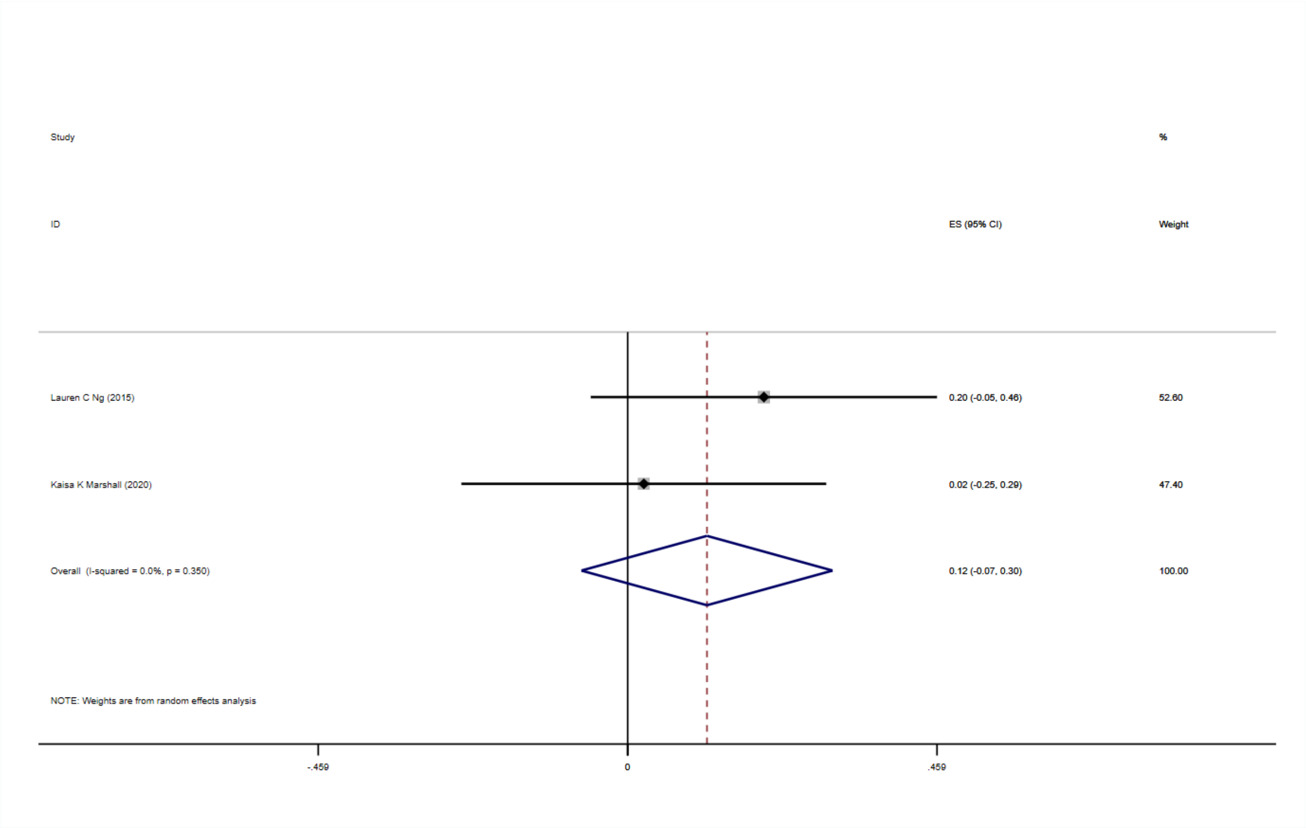

Supplementary Figure 36. touching-related words and intrusive symptoms

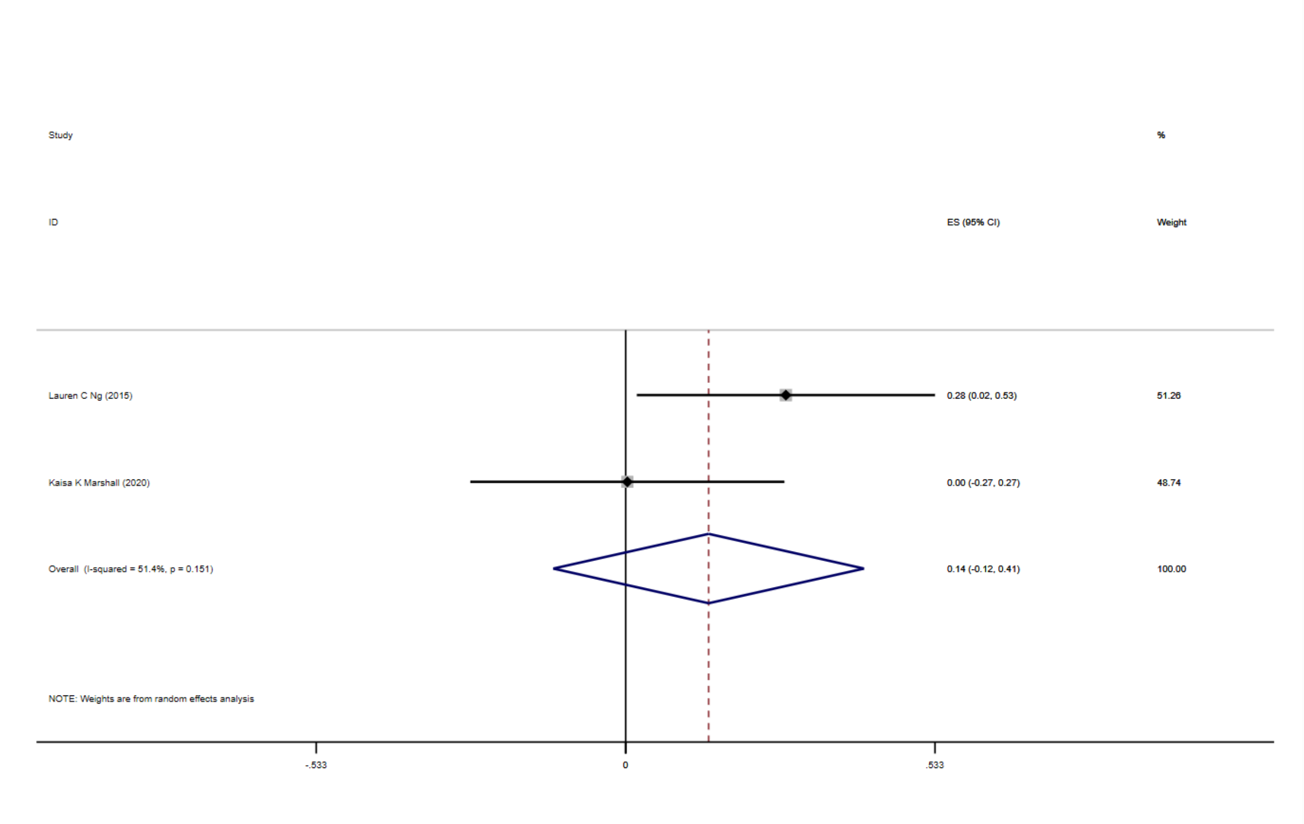

Supplementary Figure 37. hearing-related words and hyperarousal symptoms

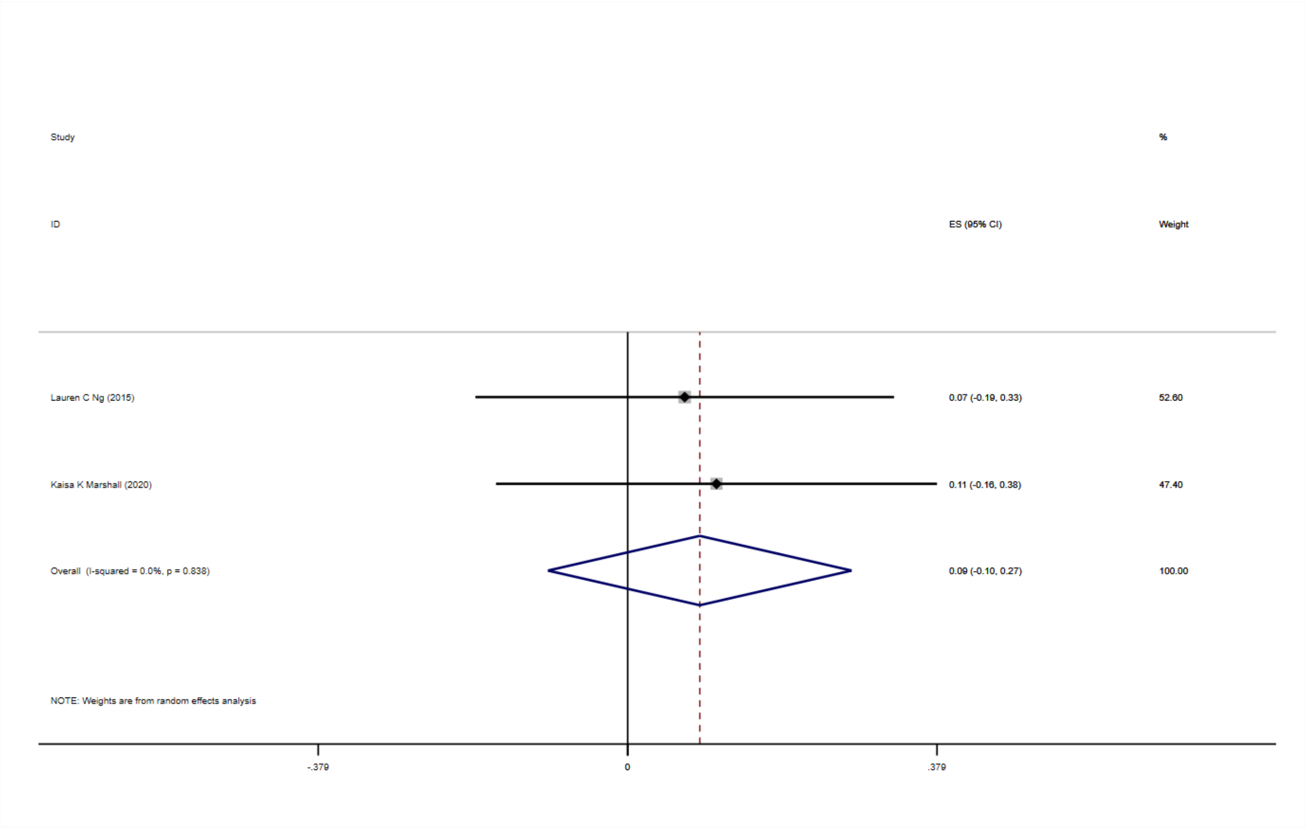

Supplementary Figure 38. hearing-related words and avoidance symptoms

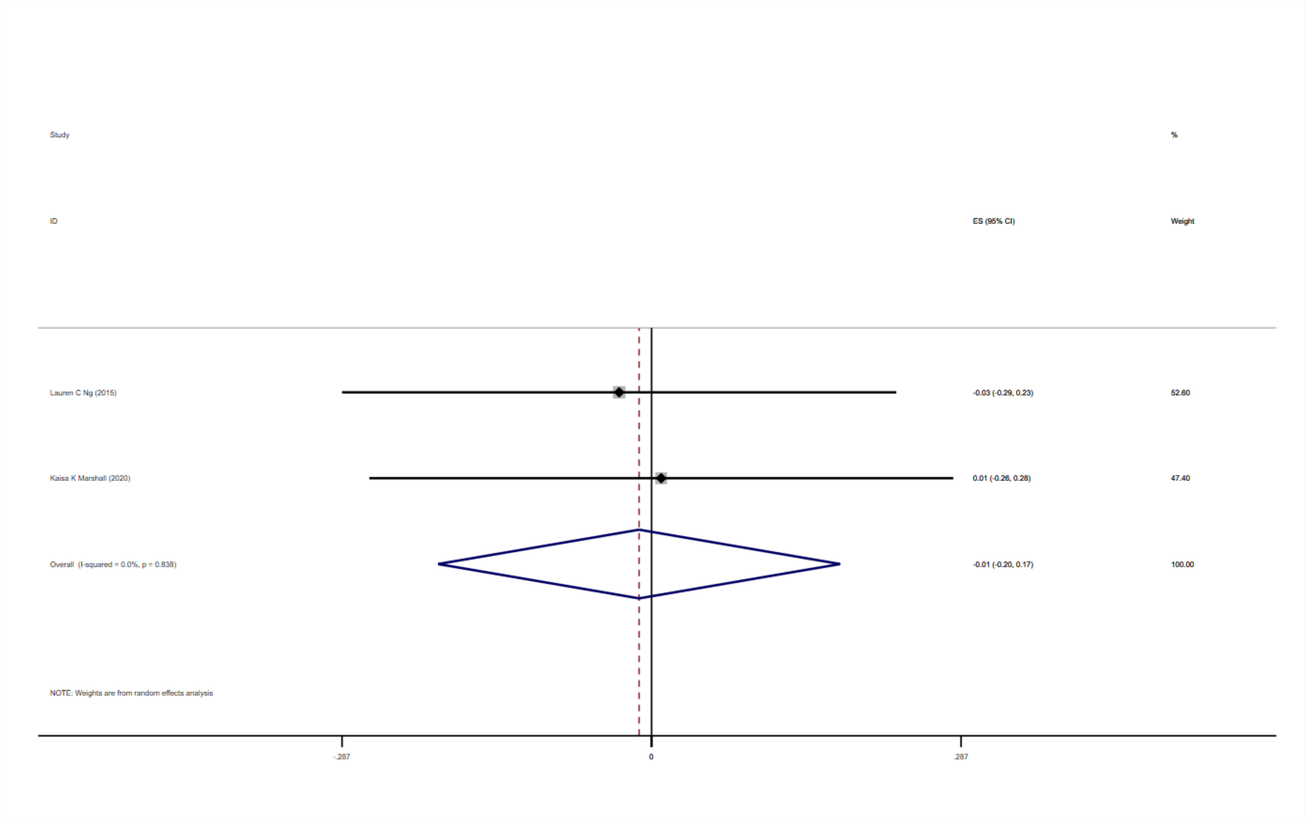

Supplementary Figure 39. hearing-related words and intrusive symptoms

1.2 Supplementary Table

	Selection				Comparability	Outcome			
Study, Year	Representativeness of the exposed cohort	Selection of the non-exposed cohort	Ascertainment of exposure	Demonstration that outcome of interest was not present at start of study	Comparability of cohorts on the basis of the design or analysis	Assessment of outcome	Was follow-up long enough for outcomes to occur	Adequacy of follow up of cohorts	Quality score
Alvarez-Conrad, 2001	★	★	★	★	★	★		★	7
D'Andrea, 2012			★	★		★	★	★	5
Miragoli, 2014	★		★	★	★	★		★	6
Ng, 2015	★		★	★	★★	★	★	★	8
Papini, 2015	★		★	★	★	★	★	★	7
Marshall, 2020	★	★	★	★	★		★	★	7
Marengo, 2022	★	★	★	★	★	★	★	★	8
Castiglioni, 2023	★	★	★	★	★★	★	★	★	9
Ellis, 2023	★	★	★	★			★	★	6
Frabetti, 2023			★	★	★★	★		★	6
Son, 2023	★	★	★	★	★★	★	★	★	9
Quillivic, 2024	★	★	★	★	★★	★	★	★	9

frontiers
